# ﻿Ophiostomatoid fungi associated with *Hylurgus ligniperda*, including six new species from eastern China

**DOI:** 10.3897/imafungus.16.169382

**Published:** 2025-10-28

**Authors:** Dan Xie, Huanwen Chen, Niya Jia, Fang Niu, Xiaomei Wang, Jia Yu, Defu Chi

**Affiliations:** 1 Key Laboratory for Sustainable Forest Ecosystem Management of Ministry of Education, College of Forestry, Northeast Forestry University, Harbin 150040, Heilongjiang, China Northeast Forestry University Harbin China; 2 National Permanent Scientific Research Base for Warm Temperate Zone Forestry of Jiulong Mountain in Beijing, Experimental Centre of Forestry in North China, Chinese Academy of Forestry, Beijing, China Experimental Centre of Forestry in North China, Chinese Academy of Forestry Beijing China

**Keywords:** *
Ceratocystiopsis
*, *
Graphilbum
*, *
Hawksworthiomyces
*, *
Leptographium
*, *
Masuyamyces
*, *
Ophiostoma
*, taxonomy

## Abstract

*Hylurgus ligniperda* is a highly successful invader among bark beetles (*Scolytinae*), and has become established in every continent where its host plants occur. Bark beetles maintain a close symbiotic relationship with ophiostomatoid fungi whose morphology is highly adapted for beetle dispersal, and the presence of these fungal symbionts actively facilitates successful bark beetle invasions. At present, the fungal community associated with *H. ligniperda* in the newly invaded eastern China is still unknown. The aims of this study were therefore to characterize the ophiostomatoid communities associated with *H. ligniperda* in China. To achieve this, a total of 435 ophiostomatoid fungal strains were isolated from 326 adult samples collected in galleries and traps. Through morphological analysis and multilocus phylogenetic approaches, 13 species across six genera (*Ceratocystiopsis*, *Graphilbum*, *Hawksworthiomyces*, *Leptographium*, *Masuyamyces*, and *Ophiostoma*) were identified, of which six species were described as new. Fungal recovery rates differed significantly between gallery-derived and trap-collected adults (*χ*² test, *p* < 0.01). Furthermore, comparative analysis of ophiostomatoid fungal communities associated with *H. ligniperda* across five continents revealed distinct and well-defined assemblage patterns in each geographical region. This study elucidates the symbiotic relationship between *H. ligniperda* and ophiostomatoid fungi during invasion, providing a theoretical foundation for further research on their cooperative invasion and colonization mechanisms.

## ﻿Introduction

Global climate change and trade globalization have significantly influenced the proliferation and distribution of forest pests, posing novel challenges for forest management. Bark beetles (*Coleoptera*, *Curculionidae*, *Scolytinae*), a highly diverse group of insects, are prevalent across global forest ecosystems (Morris et al. 2017). These beetles, which primarily target coniferous trees, exhibit strong concealment abilities and can induce extensive tree mortality, thus representing critical forest pests (Ferrenberg et al. 2014). Research indicates a strong correlation between the spread of bark beetles and climate warming (Niemiec et al. 2014). When conditions are favorable, particularly when introduced beyond their native habitats, certain species can transition from minor pests to significant threats to host trees, resulting in substantial economic losses and ecological harm (Ranger et al. 2015; Ranger 2016; Biedermann et al. 2019). In Europe, *Ips typographus* Linnaeus is regarded as the most significant pest affecting *Picea abies* (L.) H. Karst. (Schelhaas et al. 2003). Similarly, in China, the invasive alien species *Dendroctonus valens* LeConte has led to widespread mortality of *Pinus tabuliformis* Carrière, posing a serious threat to the ecological stability of forestry (Lu et al. 2009; Sun et al. 2013).

In comparison to isolated damage, the combined impact of microbial pathogens and insects can result in severe harm to forest ecosystems (Kenneth F. Raffa and Romme 2008). Consequently, it is imperative to promptly elucidate the novel interactions between forest pests and pathogens (Wingfield et al. 2016; Biedermann and Vega 2020). Ophiostomatoid fungi are primarily associated with bark beetles. These fungi exhibit similar morphological and structural traits, notably the production of adhesive spore masses, which are disseminated by bark beetles to host plants (De Beer 2013). Ophiostomatoid fungi are integral to the successful colonization of bark beetles (Hofstetter et al. 2015), significantly compromising the resistance of host trees and facilitating the invasion by these beetles (Heiniger et al. 2011). Furthermore, these fungi contribute essential nutrients for the growth and development of bark beetles within the nutrient-deficient phloem of coniferous trees (Bentz and Six 2006). Bark beetles associate with a diverse array of ophiostomatoid fungal species, including several highly pathogenic strains (Kgang et al. 2008) and staining fungi (Hýsek et al. 2021), the latter of which can adversely affect wood quality. This group of fungi are regarded as a significant threat to conifers, prompting extensive research of bark beetle fungi globally. In southeastern Australia, fourteen species of *Ophiostomatales* across six genera, along with one species of *Microascales*, have been isolated from bark beetles and infected pines (Trollip et al. 2021). In Northeast China, *Ips subelongatus* Motschulsky is associated with fourteen species of ophiostomatoid fungi spanning four genera (*Ceratocystiopsis*, *Endoconidiophora*, *Leptographium*, and *Ophiostoma*) (Wang et al. 2020). Notably, *Ophiostoma bicolor* R.W. Davidson & D.E. Wells, *O. brunneo-ciliatum* Math.-Käärik, *O. ainoae* H. Solheim, and *Grosmannia piceiperda* (Rumbold) Goid are associated with both *Ips typographus* and *Pityogenes chalcographus* Linnaeus (Repe et al. 2013).

*Hylurgus ligniperda* Fabricius (1787), commonly referred to as the red-haired pine bark beetle, is a forest insect that parasitizes the phloem of pine trees (Bedoya et al. 2019). It is presumed to be indigenous to Europe and the Mediterranean Basin, with subsequent introductions into Oceania, North America, and South America. In East Asia, *H. ligniperda* is considered an invasive species, initially invading Japan and subsequently Korea. Recently, it has successfully invaded and colonized Shandong Province in China (Lin et al. 2021). As a root-dwelling species, *H. ligniperda* typically enters its host through the soil and feeds on the phloem of sub-healthy pine trees, although it does not directly cause tree mortality. However, in newly invaded regions, the presence of *H. ligniperda* significantly affects the ecological security of local forestry systems. The invasion of *H. ligniperda* has resulted in a reduction of the windbreak and sand-fixation capacities of the coastal shelterbelt in Shandong Province, China, and its high dispersal capability poses a substantial threat to the local forest ecosystem.

Numerous studies have documented the isolation of fungi associated with *H. ligniperda*. In the forests and steppes of eastern Ukraine, *H. ligniperda* serves as a vector for diverse fungal communities (Davydenko et al. 2014). In Chile, five ophiostomatoid fungi species associated with *H. ligniperda* and *Hylastes ater* Paykull have been identified (Zhou et al. 2004c). Eight species of ophiostomatoid fungi have been isolated from *H. ligniperda* in New Zealand (Ray et al. 2006). In Poland, the ophiostomatoid fungi associated with *H. ater*, *Hylastes opacus* Erichson and *H. ligniperda* on *Pinus sylvestris* L. was examined, revealing that fungi of the genera *Grosmannia* and *Leptographium* were the most prevalent (Jankowiak and Bilański 2013). However, in the newly invaded region of *H. ligniperda* in Shandong Province, China, research of fungi associated with *H. ligniperda* remains unexplored.

In this study, we examined the ophiostomatoid fungi community associated with *H. ligniperda* in the Yanwei region of Shandong Province, China. We employed a combination of morphological observations and multilocus DNA sequence phylogenetic analysis to address existing knowledge gaps in this area. The findings of this research contribute to a deeper understanding of the ophiostomatoid fungi community linked to *H. ligniperda* in China and establish a foundation for future investigations into the occurrence and management of *H. ligniperda*.

## ﻿Materials and methods

### ﻿Collection of samples and isolation of fungi

Adults of *H. ligniperda* were collected from coastal shelterbelts in Yantai and Weihai, Shandong Province, between December 2021 and April 2023. The forest stands consisted primarily of ~45-year-old plantations of *Pinus thunbergii* Parlatore, with a minor presence of similarly aged *Pinus densiflora* Siebold & Zucc. Using sterile forceps, adults of *H. ligniperda* collected from traps and galleries were individually transferred into 2 mL sterile centrifuge tubes, transported to the laboratory, and stored at 4 °C until fungal isolation. The adults of *H. ligniperda* were subjected to disinfection for one minute using a 0.1% sodium hypochlorite (NaClO) solution, followed by three rinses with sterile water. Subsequently, the specimens were fragmented into several parts, with each part placed at a specific distance on 2% malt extract agar (MEA). All cultures were incubated in darkness at 25 °C and monitored daily. To purify the fungal isolates, hyphal tips from the fungal colonies were transferred onto 2% MEA plates. Following an initial analysis of colony and morphological characteristics, representative strains of each morphotype were selected for further studies. All strains were deposited in the Forest Protection Department of Forestry College, Northeast Forestry University, Harbin, Heilongjiang Province, China (NFF) (Table 1). Ex-type cultures of ophiostomatoid fungi described in this study are deposited in the China General Microbiological Culture Collection Center (CGMCC), and the holotype specimens (dry cultures) were deposited in the Herbarium Mycologicum, Academiae Sinicae (HMAS), Beijing, China.

**Table 1.** Representative strains of the ophiostomatoid fungi associated with adult *Hylurgus ligniperda* in Yantai and Weihai, Shandong Province, China.

Taxon	Species name^1^	Isolate numbers^2,3,4^	GenBank^5^					
			ITS	LSU	TEF-1α	βT	CAL	RPB2
1	Ceratocystiopsis pseudoweihaiensis sp. nov.	NFF1616	PQ560675	PQ550658	PQ672878	PQ672802	-	PQ672850
		NFF1617(CGMCC3.28600)^T^	PQ560676	PQ550659	PQ672879	PQ672803	-	PQ672851
2	C. pseudoyantaiensis sp. nov.	NFF1618(CGMCC3.28601)^T^	PQ560677	PQ550660	PQ672880	PQ672804	-	PQ672852
		NFF1619	PQ560678	PQ550661	PQ672881	PQ672805	-	PQ672853
3	*Graphilbum translucens*	NFF1614	PQ560671	-	PQ672874	PQ672798	PQ672822	PQ672846
		NFF1615	PQ560672	-	PQ672875	PQ672799	PQ672823	PQ672847
4	Gr. jiuguanense sp. nov.	NFF1626(CGMCC3.28602)^T^	PQ560673	-	PQ672876	PQ672800	PQ672824	PQ672848
		NFF1627	PQ560674	-	PQ672877	PQ672801	PQ672825	PQ672849
5	*Hawksworthiomyces taylorii*	NFF1628	PQ560679	PQ550662	PQ672882	PQ672806	PQ672826	PQ672854
		NFF1629	PQ560680	PQ550663	PQ672883	PQ672807	PQ672827	PQ672855
6	*Grosmannia huntii*	NFF1601	PQ560653	PQ550649	PQ672856	PQ672780	PQ672808	PQ672828
		NFF1602	PQ560654	PQ550650	PQ672857	PQ672781	PQ672809	PQ672829
7	*Leptographium koreanum*	NFF1634	PQ560655	PQ550653	PQ672858	PQ672782	PQ672810	PQ672831
		NFF1635	PQ560656	PQ550654	PQ672859	PQ672783	PQ672811	PQ672830
8	*L. radiaticola*	NFF1620	PQ560659	PQ550657	PQ672862	PQ672786	PQ672814	PQ672833
		NFF1621	PQ560658	PQ550656	PQ672861	PQ672785	PQ672813	PQ672834
		NFF1622	PQ560657	PQ550655	PQ672860	PQ672784	PQ672812	PQ672832
9	L. ligniperdae sp. nov.	NFF1605(CGMCC3.28598)^T^	PQ560660	PQ550651	PQ672863	PQ672787	PQ672815	PQ672835
		NFF1606	PQ560661	PQ550652	PQ672864	PQ672788	PQ672816	PQ672836
10	*Masuyamyces pallidulus*	NFF1664	PQ560662	-	PQ672865	PQ672789	PQ672817	PQ672837
		NFF1665	PQ560663	-	PQ672866	PQ672790	PQ672818	PQ672838
11	M. xishanensis sp. nov.	NFF1608(CGMCC3.28599)^T^	PQ560667	-	PQ672870	PQ672794	-	PQ672842
		NFF1609	PQ560668	-	PQ672871	PQ672795	-	PQ672843
12	M. dongshanensis sp. nov.	NFF1645(CGMCC3.28603)^T^	PQ560669	-	PQ672872	PQ672796	-	PQ672844
		NFF1646	PQ560670	-	PQ672873	PQ672797	-	PQ672845
13	*Ophiostoma ips*	NFF1623	PQ560664	-	PQ672867	PQ672791	PQ672819	PQ672839
		NFF1624	PQ560665	-	PQ672868	PQ672792	PQ672820	PQ672840
		NFF1625	PQ560666	-	PQ672869	PQ672793	PQ672821	PQ672841

1. Species named in black bold are novel species in this study. 2. NFF, The Forest Protection Department of Forestry College, Northeast Forestry University, Harbin, Heilongjiang Province, China. 3. CGMCC, The China General Microbiological Culture Collection. 4. T = ex-holotype isolates. 5. ITS, The internal transcribed spacer regions 1 and 2 of the nuclear ribosomal DNA operon, including the 5.8S region; LSU, The ribosomal large subunit gene region; *βT*, The β-tubulin gene region; *TEF-1α*, The transcription elongation factor-1α gene region; *CAL*, The calmodulin gene region; *RPB2*, The DNA-directed RNA polymerase II second largest subunit gene region.

### ﻿Morphological and cultural studies

The representative strains were cultured in a 2% MEA medium at 25 °C in a dark environment for a period of 7 to 15 days to facilitate morphological characterization. To examine the morphology of the newly identified species, fungal strains were first inoculated onto sterilized phloem tissues of *Pinus thunbergii* and cultured in darkness at 25 °C. Colony morphology and growth were observed daily. After 4–5 weeks, the phloem tissues were mounted on microscope slides to examine sexual or asexual structures. An Olympus BX51 (Olympus, Tokyo, Japan) microscope and a Leica Z16 APO (Leica, Leica Microsystems Ltd., Switzerland) stereomicroscope were employed to measure and photograph the microstructure of the ophiostomatoid fungi. The growth structures of each strain, including the length and width of the conidia and conidiophores, were observed and measured on microscope slides prepared with sterile water. A total of 30 measurements were conducted for the reproductive structures of each strain. The results are reported as the average (mean), standard deviation (SD), minimum (min), and maximum (max) values, presented in the format: (min –) (mean – SD) – (mean + SD) (– max).

The growth rate was assessed using the following methodology: a sterile hole punch was employed to extract a 5 mm diameter sample from the periphery of strains exhibiting robust mycelial growth. This sample was then positioned, with the mycelium side facing downward, at the center of a 100 mm Petri dish containing 25 ml of 2% malt extract agar (MEA). For each strain, five replicate plates were prepared and incubated in darkness at temperature intervals of 5 °C, ranging from 5 °C to 35 °C. Starting from the second day of incubation, the orthogonal diameters of the colonies were measured daily until the most rapidly growing mycelium reached the edge of the 2% MEA plate. Colony colors were described based on the color chart of Rayner (1970). All data related to the type specimen are deposited in MycoBank (http://www.MycoBank.org/).

### ﻿DNA extraction, PCR, and sequencing

DNA extraction was performed using an Invisorb Spin Plant Mini Kit (Tiangen, Beijing, China) from actively growing mycelia, following the manufacturer’s instructions. The internal transcribed spacer regions 1 and 2 of the nuclear ribosomal DNA operon, including the 5.8S region (ITS), the ribosomal large subunit region (LSU), the β-tubulin gene region (*βt*), the transcription elongation factor 1-α gene region (*TEF-1α*), the calmodulin gene region (*CAL*), and the DNA-directed RNA polymerase II second largest subunit gene region (*RPB2*) were subjected to PCR using the 2 × Taq PCR MasterMix (Tiangen, Beijing, China), following the manufacturer’s instructions. The primer pairs used for amplification included ITS1-F/ITS4 (Gardes and Bruns 1993)(White et al. 1990), LR0R/LR5 (Vilgalys and Hester 1990), Bt2a/Bt2b (Glass and Donaldson 1995), EF2F/EF2R (Marincowitz et al. 2015)(Jacobs et al. 2004), CL2F/CL2R (Duong et al. 2012), and Oph-RPB2F1/Oph-RPB2R1 or Oph-RPB2F2/Oph-RPB2R1 (De Beer et al. 2022). The ITS, LSU, *βT*, *TEF-1α*, and *CAL* gene regions were amplified according to the protocol described by Wang et al. (2020), and the *RPB2* gene region was amplified according to the protocol described by De Beer et al. (2022). All PCR products were sequenced by Sangon Biotech, Changchun, Jilin Province, China. All sequences obtained in this study were deposited in GenBank (Table 1) (www.ncbi.nlm.nih.gov/Genbank).

### ﻿Phylogenetic analyses

The sequences derived from the six DNA fragments were initially identified through a BLAST search in the NCBI GenBank database. Representative sequences exhibiting the highest similarity, along with model strain sequences of closely related species, were subsequently retrieved from the NCBI GenBank database. The GenBank accession numbers for these sequences are indicated on the corresponding phylogenetic trees. Sequence datasets were aligned using MAFFT v7.505 (Katoh and Standley 2013) and trimmed at both termini as necessary. To eliminate ambiguously aligned regions, all datasets were processed using Gblocks 0.91b (Talavera and Castresana 2007) with less stringent parameters. PhyloSuite v1.2.3 (Zhang et al. 2020) was employed to concatenate the datasets of individual gene regions obtained from Gblocks. Subsequently, PhyloSuite v1.2.3 facilitated the construction of multilocus phylogenetic trees using Maximum Likelihood (ML) and Bayesian Inference (BI) methods.

The ModelFinder v2.2.0 (Kalyaanamoorthy et al. 2017) was used to select the best-fit partition model (Edge-linked) using Akaike information criterion (AIC) (Table 2). Maximum likelihood (ML) phylogenies were inferred using IQ-TREE v2.2.0 (Nguyen et al. 2014) under Edge-linked partition model for 1000 standard bootstraps, as well as the Shimodaira-Hasegawa-like approximate likelihood-ratio test. Bayesian Inference (BI) phylogenies were inferred using MrBayes v3.2.7a (Ronquist et al. 2012) under partition model (2 parallel runs, 5000000 generations), until the average standard deviation of split frequencies was < 0.01. Trees were sampled every 100 generations, in which the initial 25% of sampled data were discarded as burn-in. The remaining trees were used to calculate the posterior probabilities. The phylogenetic tree was edited in FigTree v.1.4.3 (http://tree.bio.ed.ac.uk/software/figtree/) and Adobe Illustrator CS6. The final alignment results and the derived topology were archived in TreeBASE (No. 31864).

**Table 2.** Nucleotide substitution models obtained from ModelFinder v2.2.0.

Genus/Other level	Dataset (Positions)	Nucleotide substitution models	
		IQ-TREE	Mrbayes
	ITS complex (438 bp)	GTR + F + I + G4	GTR + F + I + G4
	LSU complex (800 bp)	GTR + F + I + I + R2	GTR + F + I + G4
* Ceratocystiopsis *	ITS (627 bp)	GTR + F + I + I + R2	GTR + F + G4
	*βT* (407 bp)	TIM2 + F + I + G4	GTR + F + I + G4
	*TEF1-α* (572 bp)	TN + F + G4	GTR + F + G4
	ITS + *βT* + *TEF1-α* (1606 bp)	ITS: GTR+F+I+I+R2; *βT* + *TEF1-α*: TIM2 + F + I + G4	ITS: GTR + F + G4; *βT* + *TEF1-α*: GTR + F + I + G4
* Graphilbum *	ITS (621 bp)	SYM + G4	SYM + G4
	*TEF1-α* (543 bp)	TN + F + I + G4	GTR + F + I + G4
	*CAL* (604 bp)	GTR + F + I + G4	GTR + F + I + G4
	ITS + *TEF1-α* + CAL (2102 bp)	ITS: TIMe + R2; *TEF1-α*: TN + F + I + G4; *CAL*: GTR + F + I + G4	ITS: SYM + G4; *TEF1-α*: GTR + F + I + G4; *CAL*: GTR + F + I + G4
* Hawksworthiomyces *	ITS + *TEF1-α* + *RPBII* (1768 bp)	ITS: TIM + F + G4; *TEF1-α*: TIM2 + F + I; *RPBII*: TIM3 + F + G4	ITS: GTR + F + G4; *TEF1-α*: GTR + F + I; *RPBII*: GTR + F + G4
* Leptographium *	*βT* + *TEF1-α* (778 bp)	*βT*: TN + F + R2 *TEF1-α*: TIM2 + F + I + G4	*βT*: GTR + F + G4 *TEF1-α*: GTR + F + I + G4
*L. olivaceum* complex	*βT* (277 bp)	TN + F + R2	HKY + F + G4
	*TEF1-α* (582 bp)	TIM3 + F + G4	GTR + F + G4
	*CAL* (412 bp)	TIM3 + F + G4	GTR + F + G4
*Ophiostoma ips* complex and *Masuyamyces*	ITS (461 bp)	GTR + F + I + I + R2	GTR + F + G4
	*βT* (277 bp)	TIM3 + F + R4	GTR + F + G4

### ﻿Statistical analysis

The isolation frequencies of fungal taxa common to adults obtained from galleries and traps were compared. Statistical significance was determined using a chi-square test in SPSS Statistics 20.0 (SPSS Inc.).

## ﻿Results

### ﻿Collection of samples and isolation of fungi

In this study, 435 strains of ophiostomatoid fungi were isolated from 326 adult beetles. Preliminary identification was conducted based on growth rate and macroscopic and microscopic morphological characteristics. All strains were preserved and DNA extracted. Standard nucleotide BLAST searches at GenBank were performed using the ITS and LSU sequences of all strains for preliminary classification and affinity assessment. Subsequently, 28 representative strains were selected for comprehensive morphological examination and multilocus phylogenetic analysis.

### ﻿Phylogenetic analysis

**Figure 1.**
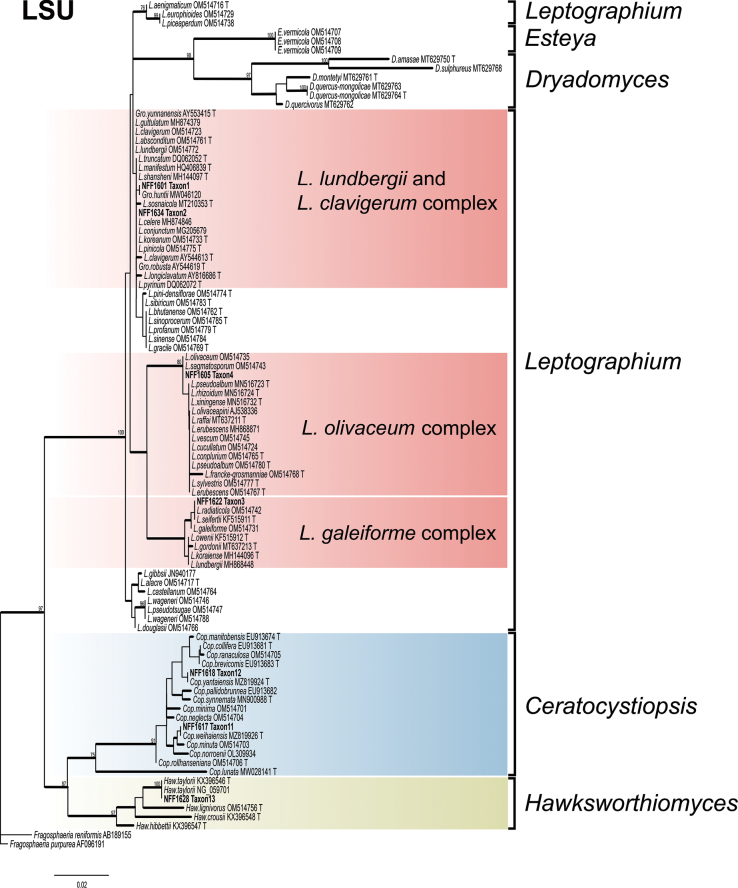
Maximum Likelihood phylogeny of LSU region for isolates residing in *Leptographium*, *Ceratocystiopsis*, and *Hawksworthiomyces*. Sequences generated in this study are printed in bold. Bold branches indicate posterior probability values ≥ 0.9, while ML bootstrap values of ≥ 75% are recorded at nodes. T = ex-type cultures.

**Figure 2.**
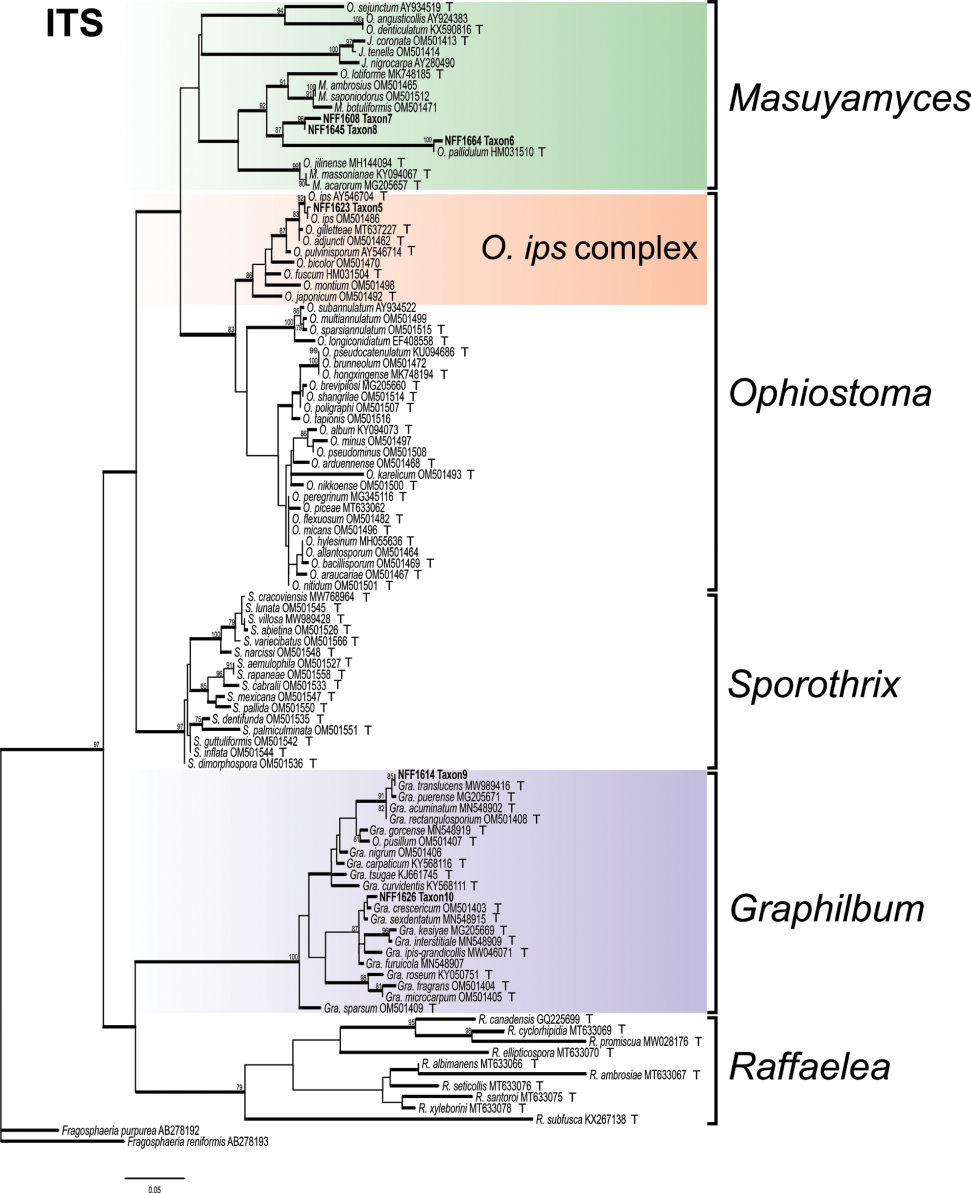
Maximum Likelihood phylogeny of ITS region for isolates residing in *Masuyamyces*, *Ophiostoma*, and *Graphilbum*. Sequences generated in this study are printed in bold. Bold branches indicate posterior probability values ≥ 0.9, while ML bootstrap values of ≥ 75% are recorded at nodes. T = ex-type cultures.

Phylograms generated by ML are presented for all datasets, with branch supports indicated from both ML and BI analyses. The phylogenetic analysis at the genus level was first conducted based on the ITS and LSU regions (Schoch et al. 2012), followed by a more precise species-level delineation utilizing additional gene regions (namely *βT*, *TEF1-α*, *CAL*, and *RPB2*) and their concatenated dataset (De Beer et al. 2016a; De Beer et al. 2022). The phylogenetic analysis revealed that 28 representative isolates were distributed across 13 terminal clades or phylogenetic groups, spanning six genera within the *Ophiostomatales* (Figs 1, 2). The results of the phylogenetic analysis are presented as follows: *Ceratocystiopsis* (Taxa 1–2), *Graphilbum* (Taxa 3–4), *Hawksworthiomyces* (Taxon 5), *Leptographium* (Taxa 6–9), *Masuyamyces* (Taxa 10–12), and *Ophiostoma* (Taxon 13).

#### ﻿*Ceratocystiopsis*

**Figure 3.**
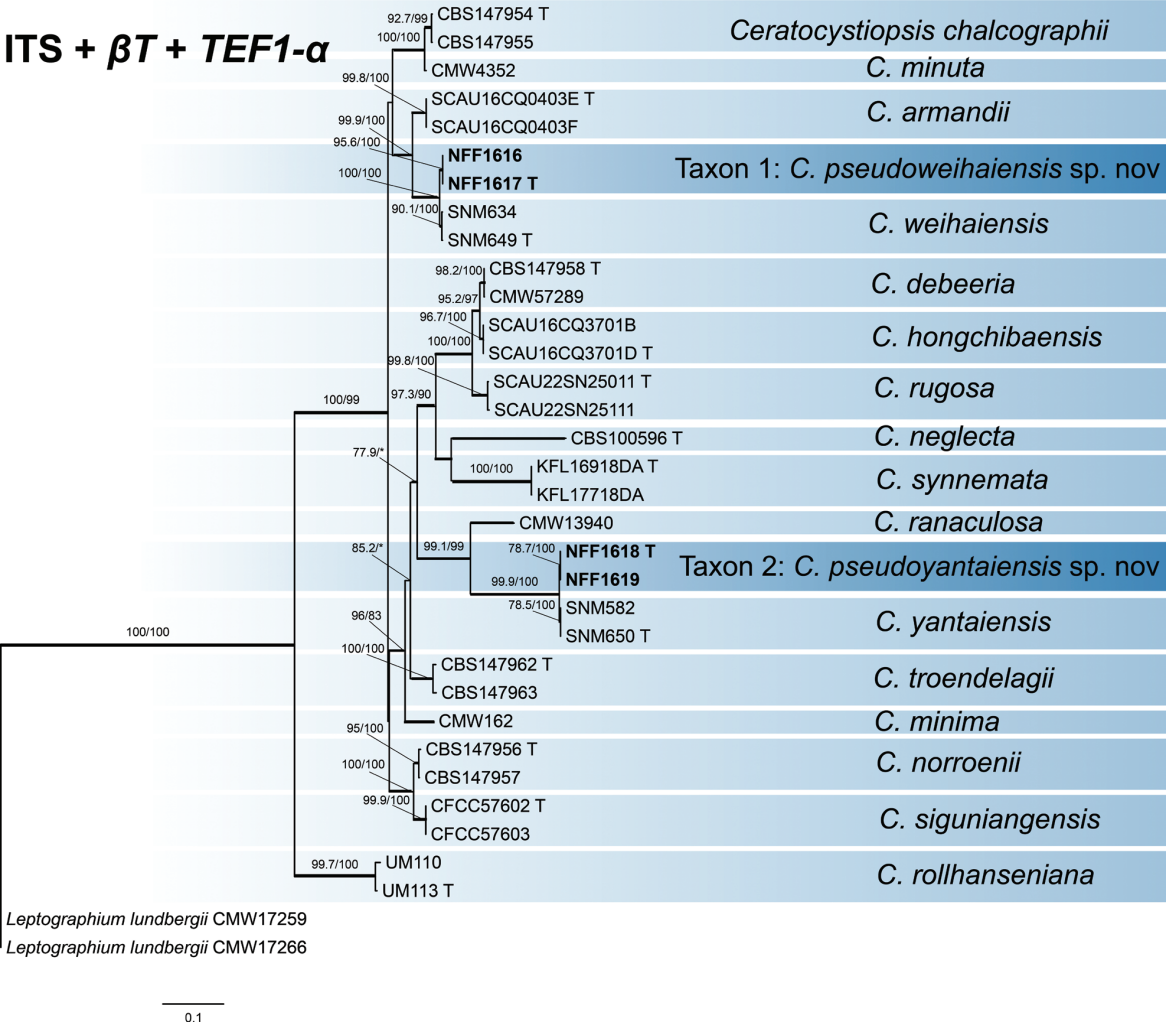
Maximum Likelihood tree of *Ceratocystiopsis* generated from the combined (ITS + *βT* + *TEF1-α*) sequence data. Sequences generated in this study are printed in bold. Bold branches indicate posterior probability values ≥ 0.9, while ML SH-aLRT/UFBooT values of ≥ 75% are recorded at nodes, < 75% are indicated by the symbol *. T = ex-type cultures.

Phylogenetic inferences were constructed using ITS (627 positions), *βT* (407 positions), *TEF1-α* (572 positions), and the concatenated datasets (ITS + *βT* + *TEF1-α*, 1606 positions). Our four representative isolates formed two distinct, well-supported terminal clades based on the ITS + *βT* + *TEF1-α* phylogenetic tree (Fig. 3), which are interpreted as two distinct taxa, each as representing an undescribed taxon. The two representative strains of Taxon 1 are most closely related to the *C. weihaiensis* R.L. Chang & X.Y. Zhang strains of the known species, while the two representative strains of Taxon 2 are closely related to the *C. yantaiensis* R.L. Chang & X.Y. Zhang strains. Both *βT*- and *TEF1-α*-based phylogenies robustly resolved Taxon 1 and Taxon 2 as distinct from related species (Suppl. materials 4, 5).

#### ﻿*Graphilbum*

**Figure 4.**
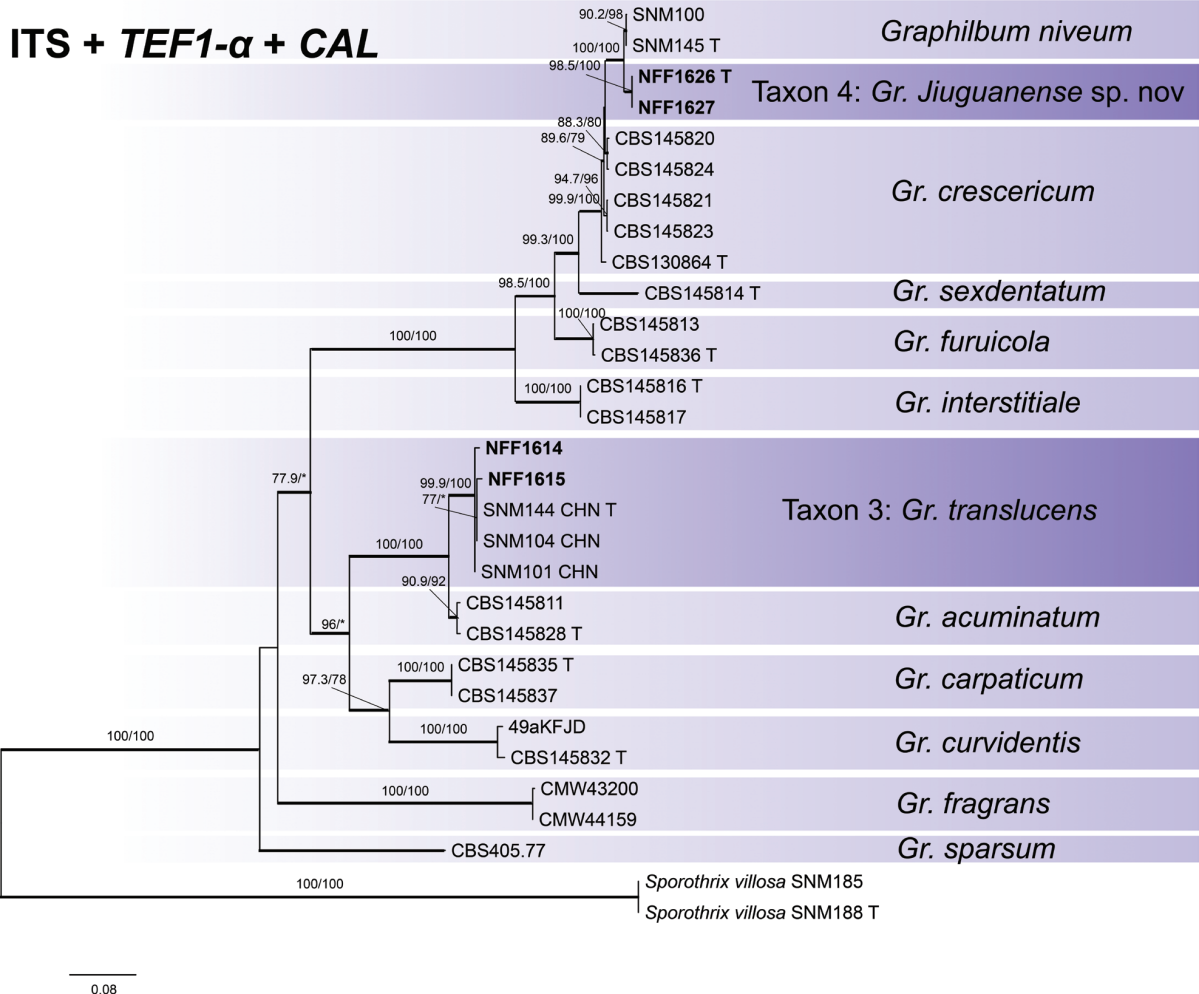
Maximum Likelihood tree of *Graphilbum* generated from the combined (ITS + *TEF1-α* + *CAL*) sequence data. Sequences generated in this study are printed in bold. Bold branches indicate posterior probability values ≥ 0.9, while ML SH-aLRT/UFBooT values of ≥ 75% are recorded at nodes, < 75% are indicated by the symbol *. T = ex-type cultures.

Phylogenetic inferences were constructed using ITS (621 positions), *TEF1-α* (543 positions), *CAL* (604 positions), and the concatenated datasets (ITS + *TEF1-α* + *CAL*, 2102 positions). Individual gene trees and the concatenated phylogeny congruently elucidated interspecific relationships within the genus (Fig. 4, Suppl. materials 6–8). Based on the ITS + *TEF1-α* + *CAL* phylogenetic tree, four representative strains were categorized into two distinct branches (Fig. 4). Taxon 3 represents *Gr. translucens* R.L. Chang & X.Y. Zhang, which were grouped in an independent clade with *Gr. acuminatum* R. Jankowiak & H. Solheim. Whereas Taxon 5 could not be equated to a known species, which is most closely related to the known species *Gr. niveum* R.L. Chang & X.Y. Zhang.

#### ﻿*Hawksworthiomyces*

**Figure 5.**
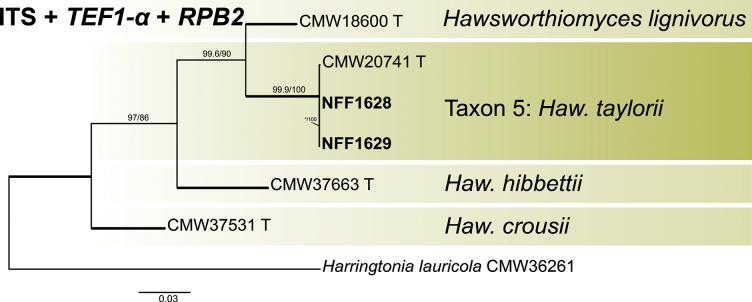
Maximum Likelihood tree of *Hawksworthiomyces* generated from the combined (ITS + *TEF1-α* + *RPB2*) sequence data. Sequences generated in this study are printed in bold. Bold branches indicate posterior probability values ≥ 0.9, while ML SH-aLRT/UFBooT values of ≥ 75% are recorded at nodes, < 75% are indicated by the symbol *. T = ex-type cultures.

In *Hawksworthiomyces*, the concatenated datasets (ITS + *TEF1-α* + *RPB2*, 1768 positions) were used to construct the phylogenetic inferences. The two representative strains of Taxon 5 were clustered with the type strains of *H.w. taylorii* Z.W. de Beer, Marincowitz & M.J. Wingfield and had a high support rate (Fig. 5). Accordingly, that strain of the Taxon 5 was confirmed as *H.w. taylorii*.

#### ﻿*Leptographium*

**Figure 6.**
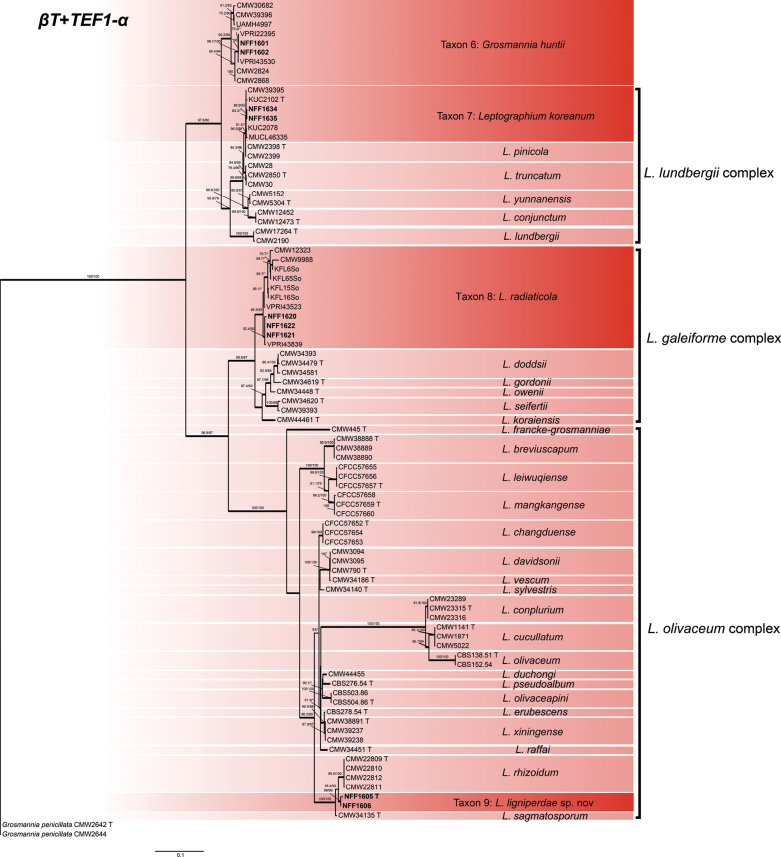
Maximum Likelihood tree of *Leptographium* generated from the combined (*βT* + *TEF1-α*) sequence data. Sequences generated in this study are printed in bold. Bold branches indicate posterior probability values ≥ 0.9, while ML SH-aLRT/UFBooT values of ≥ 75% are recorded at nodes, < 75% are indicated by the symbol *. T = ex-type cultures.

The phylogenetic tree constructed using LSU sequences (Fig. 1) indicated that the representative strains within *Leptographium* were divided into three assemblage species. In *Leptographium*, the concatenated datasets (*βT* + *TEF1-α*, 778 positions) was used to construct phylogenetic inference. The results showed that our nine representative isolates were nested into four robust clades (Fig. 6). Phylogenetic analysis of this combined dataset confirmed that Taxon 6, together with southeastern Australian strains (VPRI22395 and VPRI43530) (Trollip et al. 2021), clustered within the same subclade of *Grosmannia huntii* (Rob.-Jeffr.) Zipfel, Z.W. de Beer & M.J. Wingf., demonstrating their high similarity with strong statistical support (SH-aLRT/UFBoot = 99.7/100). Taxon 7 was situated within the *L. lundbergii* complexes, which represents *L. koreanum* J.J. Kim & G.H. Kim, grouped with *L. pinicola* (K. Jacobs & M.J. Wingf.) Z.W. de Beer in a distinct lineage. Taxon 8 is positioned within the *L. galeiforme* complex. The *βT* + *TEF1-α* phylogeny resolved the three isolates of Taxon 8 form a monophyletic group with strong support (SH-aLRT/UFBoot = 92.4/93) alongside Australian strains (VPRI43839) representing *L. radiaticola* (J.J.Kim, Seifert & G.H.Kim) M.Procter & Z.W.de Beer, confirming their high degree of similarity and clustering in southeast Australia strains. The concatenated datasets phylogenetic analysis reveals that Taxon 9 forms a distinct, well-supported clade within the *L. olivaceum* complex, representing a novel, undescribed species. Taxon 9 formed a clade with *L. rhizoidum* M.L. Yin, Z.W. de Beer & M.J. Wingf. and *L. sagmatosporum* (E.F. Wright & Cain) M.L. Yin, Z.W. de Beer & M.J. Wingf. Phylogenetic analysis of the *L. olivaceum* complex was performed based on the *βT* (277 positions), *TEF1-α* (582 positions), and *CAL* (412 positions) datasets. The *βT* and *CAL* tree revealed clearer phylogenetic relationships among the species in the *L. olivaceum* complex than in the *TEF1-α* trees (Suppl. materials 9–11).

### ﻿*Masuyamyces* and *Ophiostoma ips* complex

**Figure 7.**
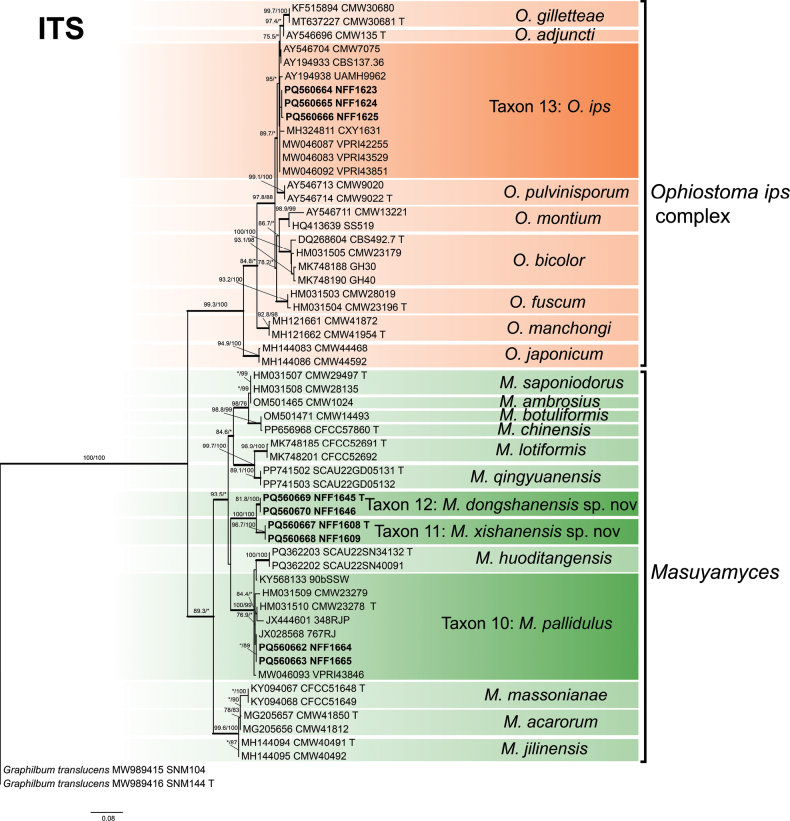
Maximum Likelihood tree of *Masuyamyces* and *Ophiostoma ips* complex generated from the ITS sequence data. Sequences generated in this study are printed in bold. Bold branches indicate posterior probability values ≥ 0.9, while ML SH-aLRT/UFBooT values of ≥ 75% are recorded at nodes, < 75% are indicated by the symbol *. T = ex-type cultures.

In *Masuyamyces* and *Ophiostoma ips* complex, the ITS (461 positions) and *βT* (277 positions) datasets were used to construct the phylogenetic inferences. The results showed that our nine representative isolates nested into four robust clades (Fig. 7, Suppl. material 12), well-supported terminal clades, representing *O. ips* (Rumb.) Nannf. (Taxon 13), *M. pallidulus* (Linnakoski, Z.W. de Beer & M.J. Wingf.) Z.W. de Beer & M. Procter (Taxon 10) and two undescribed taxa (Taxa 11 and 12). Apart from *O. ips*, *M. pallidulus* and two undescribed taxa belonged to *Masuyamyces*. Our three isolates of *O. ips* and two isolates of *M. pallidulus* all formed a poorly supported subclade, somewhat divergent from the other strain. Taxa 11 and 12 were sister species, forming a well-supported clade (Fig. 7, Suppl. material 12).

### ﻿Taxonomy

In this study, 13 phylogenetic species were identified, of which six were confirmed to represent different terminal branches, including two phylogenetic species of *Ceratocystiopsis*, one phylogenetic species of *Graphilbum*, one phylogenetic species of *Leptographium* and two phylogenetic species of *Masuyamyces*. They are interpreted as new species of *Ophiostomatales*.

#### ﻿ Ceratocystiopsis pseudoweihaiensis

Taxon classificationAnimaliaOphiostomatalesOphiostomataceae

D. Xie, H. W. Chen & D. F. Chi sp. nov.

4AA08C99-586B-5AA3-805E-4771CD3F9623

856956

Fig. 8

##### Etymology.

The epithet *pseudoweihaiensis* (Latin) refers to the morphological resemblance and phylogenetic affinities with *Ceratocystiopsis weihaiensis*.

##### Diagnosis.

*Ceratocystiopsis pseudoweihaiensis* is phylogenetically distinct from all morphologically similar species, from which it can be readily distinguished using molecular sequence data for the beta-tubulin (*βT*) and the elongation factor 1-alpha (*TEF1-α*) regions (Suppl. materials 4, 5).

##### Type.

**China** • Shandong Province: Yantai City, from *Hylurgus ligniperda*, Nov. 2022, Dan Xie (***holotype***HMAS 354192, dried culture prepared from NFF1617; ex-holotype culture CGMCC3.28600 = NFF1617).

##### Description.

***Sexual morph*** not observed. ***Asexual morph*** observed mononematous. ***Mononematous morph***: hyalorhinocladiella-like, ***conidiogenous cells*** arising directly from mycelium, (3.5–)6–52(–87) × (0.5–)1–1.5(–2) μm; ***conidia*** hyaline, smooth, unicellular short oblong, with rounded ends or clavate, (1.5–)2–3(–3.5) × (0.5–)1–1.5(–2) µm.

##### Culture characteristics.

The colonies are white in color on 2%MEA, hyphae submerged in agar with aerial mycelium. The optimal temperature for growth is 25–30 °C, reaching 63 mm diam in 15 days. Slow growth at 5 °C and 35 °C, growth diameter of 11 mm and 15 mm in 15 days.

**Figure 8.**
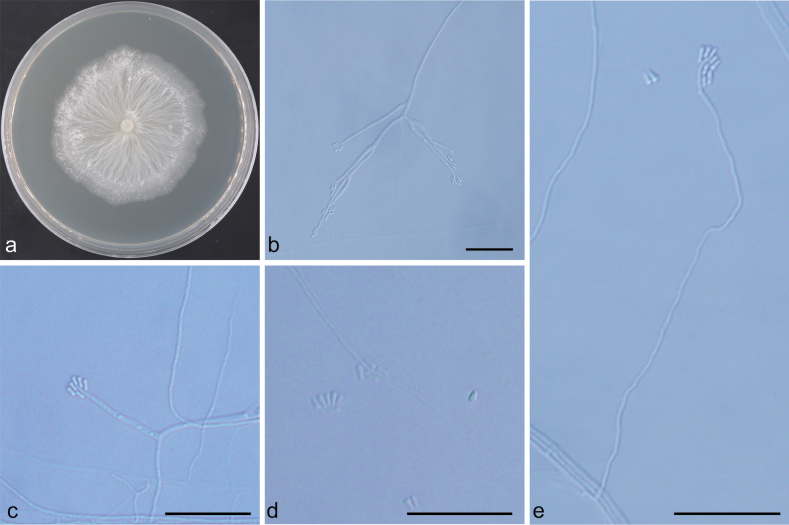
Morphological characteristics of *Ceratocystiopsis pseudoweihaiensis* sp. nov. (NFF1617). a. Fifteen-day-old culture on 2% MEA; b–e. Hyalorhinocladiella-like asexual morph: conidiogenous cells and conidia. Scale bars: 20 μm (b–e).

##### Ecology.

Isolated from migratory beetles living in *Pinus* hosts. Insect vector: *H. ligniperda*.

##### Distribution.

Currently known only from Shandong Province, China.

##### Additional specimens examined.

**China** • Shandong Province: Yantai City, from *H. ligniperda*, Nov. 2022, D. Xie (living culture NFF1616).

##### Notes.

Phylogenetic analyses showed that *C. pseudoweihaiensis* is a close relative of *C. weihaiensis*. However, several morphological differences separate them. *Ceratocystiopsis weihaiensis* produces light brown colonies, while those of *C. pseudoweihaiensis* are white. The key distinguishing feature is the length of the conidiogenous cells, which are significantly longer in *C. pseudoweihaiensis* (6–52 μm) than in *C. weihaiensis* (10.9–29.2 μm) (Chang et al. 2021). Based on both phylogenetic and morphological evidence, we propose *C. pseudoweihaiensis* as a novel species.

#### ﻿ Ceratocystiopsis pseudoyantaiensis

Taxon classificationAnimaliaOphiostomatalesOphiostomataceae

D. Xie, H. W. Chen & D. F. Chi sp. nov.

36A7FFF5-F80D-547E-9F8D-0764AB136E16

856957

Fig. 9

##### Etymology.

The epithet *pseudoyantaiensis* (Latin) refers to the morphological resemblance and phylogenetic affinities with *Ceratocystiopsis yantaiensis*.

##### Diagnosis.

*Ceratocystiopsis pseudoyantaiensis* is phylogenetically distinct from all morphologically similar species, from which it can be readily distinguished using molecular sequence data for the beta-tubulin (*βT*) and the elongation factor 1-alpha (*TEF1-α*) regions (Suppl. materials 4, 5).

##### Type.

**China** • Shandong Province: Yantai City, from *H. ligniperda*, Nov. 2022, Dan Xie (**holotype**HMAS 354193, dried culture prepared from NFF1618; ex-holotype culture CGMCC3.28601 = NFF1618).

**Figure 9.**
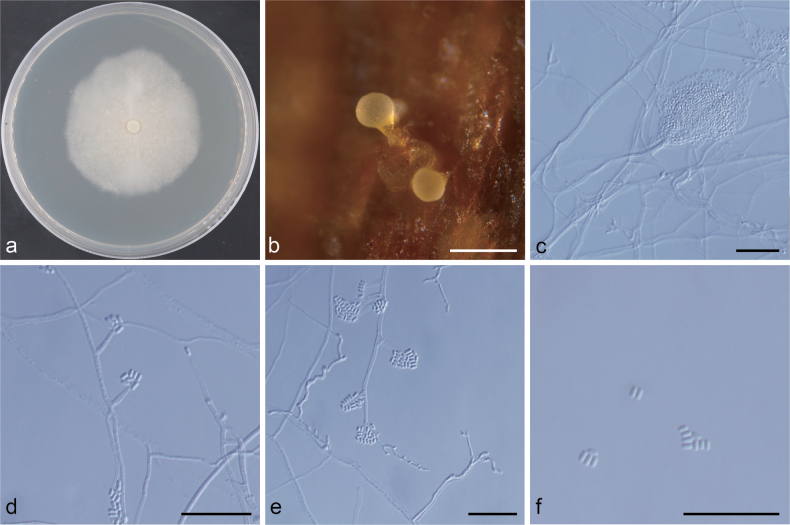
Morphological characteristics of *Ceratocystiopsis pseudoyantaiensis* sp. nov. (NFF1618). a. Fifteen-day-old culture on 2% MEA; b, c. Pesotum-like asexual morph; d–f. Hyalorhinocladiella-like asexual morph: conidiogenous cells and conidia. Scale bars: 100 μm (b); 20 μm (d–f).

##### Description.

***Sexual morph*** not observed. ***Asexual morphs*** observed both synnematous and mononematous. ***Synnematous morph***: pesotum-like, (47–)61–183(–283) μm long including ***conidiogenous*** apparatus, curving, faint yellow, the base dark yellow, aggregating into a mucilaginous spore drop, (36–)35–95(–165) μm in width. ***Mononematous morph***: hyalorhinocladiella-like, ***conidiogenous cells*** arising directly from mycelium, (2.5–)4–24(–42) × (0.5–)1–1.5(–2) µm; ***conidia*** hyaline, smooth, unicellular, short oblong, measuring (1.5–)2–2.5(–3) × (0.5–)1–1.5(–2) μm.

##### Culture characteristics.

The colonies are faint yellow in color on 2%MEA, hyphae submerged in agar with aerial mycelium. The optimal temperature for growth is 30 °C, reaching 79 mm diam in 15 days. Growth slower at 5 °C, 14 mm diam in 15 days.

##### Ecology.

Isolated from migratory beetles living in *Pinus* hosts. Insect vector: *H. ligniperda*.

##### Distribution.

Currently known only from Shandong Province, China.

##### Additional specimens examined.

**China** • Shandong Province: Yantai City, from *H. ligniperda*, Nov. 2022, D. Xie (living culture NFF1619).

##### Notes.

Phylogenetic analyses showed that *C. pseudoyantaiensis* is phylogenetically closely related to *C. yantaiensis*. Morphologically, *C. pseudoyantaiensis* differs from *C. yantaiensis* in its conidiogenous cells; those of *C. yantaiensis* are branched, while those of *C. pseudoyantaiensis* are not (Chang et al. 2021). Based on both phylogenetic and morphological evidences, we propose the recognition of *C. pseudoyantaiensis* as a novel species.

#### ﻿ Graphilbum jiuguanense

Taxon classificationAnimaliaOphiostomatalesOphiostomataceae

D. Xie, H. W. Chen & D. F. Chi sp. nov.

40AC1594-7EB3-5448-A795-B2D1E7F5A7E6

856955

Fig. 10

##### Etymology.

The epithet *jiuguanense* (Latin) refers to the Jiu Guan village from where this taxon was first isolated.

##### Diagnosis.

*Graphilbum jiuguanense* is phylogenetically distinct from all morphologically similar species, from which it can be readily distinguished using molecular sequence data for the ITS, the elongation factor 1-alpha (*TEF1-α*) and the calmodulin (*CAL*) regions (Suppl. materials 6–8).

##### Type.

**China** • Shandong Province: Yantai City, from *H. ligniperda*, Nov. 2022, Dan Xie (***holotype***HMAS 354190, dried culture prepared from NFF1626; ex-holotype culture CGMCC3.28602 = NFF1626).

##### Description.

***Sexual morph*** not observed. ***Asexual morphs*** observed both synnematous and mononematous. ***Synnematous morph***: pesotum-like, (109–)179–309(–358) μm long including ***conidiogenous*** apparatus, curving, the base dark yellow, (32–)42–80(–141) μm in width. ***Conidiogenous cells*** hyaline, (14–)18–44(–53) × (1–)2–2.5(–3) μm. ***Conidia*** hyaline, one-celled, cylindrical to obovoid, (3–)4–5.5(–6) × (1–)1.5–2(–3) μm. ***Mononematous morph***: hyalorhinocladiella-like, ***conidiogenous cells*** arising directly from mycelium, (9–)24–57(–92) × (0.5–)1–1.5(–2) µm; ***conidia*** hyaline, single-celled, smooth, ellipsoidal to ovoid, (2.5–)3–5(–6) × (1–)1.5–2(–2.5) μm.

**Figure 10.**
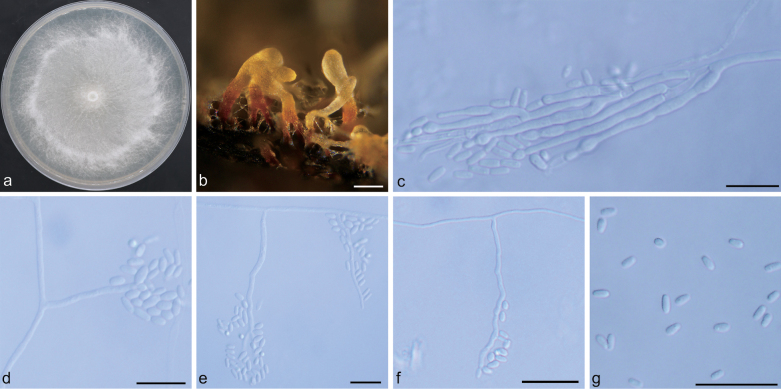
Morphological characteristics of *Graphilbum jiuguanense* sp. nov. (NFF1626). a. Ten-day-old culture on 2% MEA; b, c. Pesotum-like asexual morph; d–g. Hyalorhinocladiella-like asexual morph: conidiogenous cells and conidia. Scale bars: 100 μm (b); 10 μm (c–e); 20 μm (f, g).

##### Culture characteristics.

Colonies on 2% MEA medium fast growing in the dark, hyphae submerged in agar with aerial mycelium, mycelial growing disorderly, white. The optimal temperature for growth is 30 °C, reaching 86 mm diam in 7 days. Growth slower at 35 °C, 7 mm diam in 7 days.

##### Ecology.

Isolated from migratory beetles living in *Pinus* hosts. Insect vector: *H. ligniperda*.

##### Distribution.

Currently known only from Shandong Province, China.

##### Additional specimens examined.

**China** • Shandong Province: Yantai City, from *H. ligniperda*, Nov. 2022, D. Xie (living culture NFF1627).

##### Notes.

Phylogenetic analyses show that *Gr. jiuguanense* is phylogenetically close to *Gr. niveum*. Morphologically, the colony of *Gr. niveum* is light brown, while that of *Gr. jiuguanense* is white with a disorganized mycelial growth pattern. Furthermore, *Gr. jiuguanense* produces both pesotum-like and hyalorhinocladiella-like asexual morphs, whereas *Gr. niveum* exclusively exhibits the hyalorhinocladiella-like morph (Chang et al. 2021). Based on both phylogenetic and morphological evidence, we propose the recognition of *Gr. jiuguanense* as a novel species.

#### ﻿ Leptographium ligniperdae

Taxon classificationAnimaliaOphiostomatalesOphiostomataceae

D. Xie, H. W. Chen & D. F. Chi sp. nov.

7B556177-85EA-5C11-A927-8BD96DF738F4

856952

Fig. 11

##### Etymology.

The epithet *ligniperdae* (Latin) refers to the insect of *Hylurgus ligniperda*, the bark beetle vector of this species.

##### Diagnosis.

*Leptographium ligniperdae* is phylogenetically distinct from all morphologically similar species, from which it can be readily distinguished using molecular sequence data for the beta-tubulin (*βT*), the elongation factor 1-alpha (*TEF1-α*), and the calmodulin regions (*CAL*) (Suppl. materials 9–11).

##### Type.

**China** • Shandong Province: Yantai City, from *H. ligniperda* infesting *Pinus thunbergii*, Apr.2022, Dan Xie (***holotype***HMAS 354189, dried culture prepared from NFF1605; ex-holotype culture CGMCC3.28598 = NFF1605).

##### Description.

***Sexual morph*** not observed. ***Asexual morphs*** observed both synnematous and mononematous. ***Synnematous morph***: pesotum-like, (110–)200–310(–356) μm long including ***conidiogenous*** apparatus, the base dark brown, expanding branches at the apex, (22–)28–58(–78) μm in width. ***Conidiogenous cells*** hyaline, cylindrical, (7–)9–16(–19) × 1–2.5 μm. ***Conidia*** hyaline, one-celled, cylindrical to obovoid, (5–)6.5–8 (–10) × (2.5–)3–3.5(–4) μm. ***Mononematous morph***: leptographium-like, arising directly from mycelium; ***conidiophores***, simple to strongly branched, hyaline, (41–)43–127(–226) µm long; ***conidiogenous cells*** (6–)8–18(–26) μm long; ***conidia*** hyaline, single-celled, smooth, oblong, (3.4–)4.8–7.6(–9.4) × (1.3–)1.7–2.5(–3) μm.

##### Culture characteristics.

Colonies on 2% MEA medium fast growing in the dark, reaching 88 mm in diam. in 7 days at 25 °C, growth rate up to 15 mm/day at the fastest. Hyphae submerged in agar with aerial mycelium, dark-olivaceous to brown. Optimal growth temperature 25–30 °C, no growth observed at 5 °C and 35 °C.

**Figure 11.**
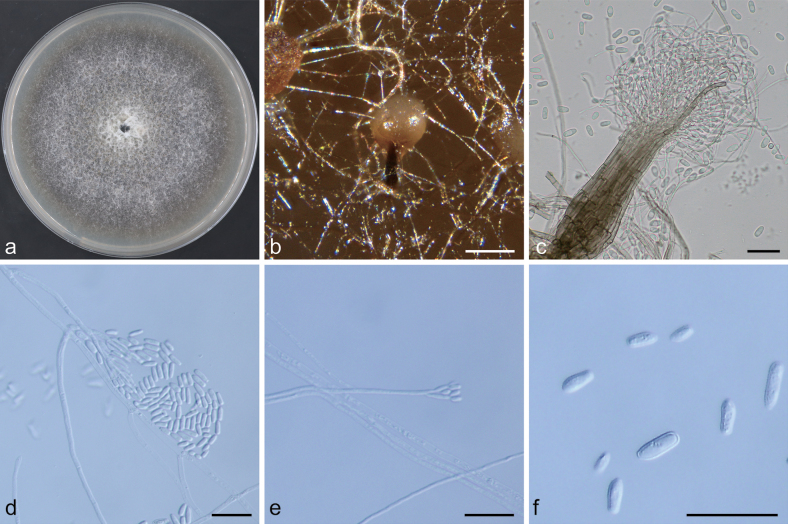
Morphological characteristics of *Leptographium ligniperdae* sp. nov. (NFF1605). a. Ten-day-old culture on 2% MEA; b, c. Pesotum-like asexual morph; d–f. Leptographium-like asexual morph: conidiogenous cells and conidia. Scale bars: 200 μm (b); 20 μm (c–f).

##### Ecology.

Isolated from beetles found on *Pinus* hosts. Host trees: *Pinus thunbergii* and *Pinus densiflora*. Insect vector: *H. ligniperda*.

##### Distribution.

Currently known only from Shandong Province, China.

##### Additional specimens examined.

**China** • Shandong Province: Yantai City, from *H. ligniperda* infesting *Pinus densiflora*, Apr.2022, D. Xie (living culture NFF1606).

##### Notes.

*Leptographium ligniperda* belongs to the *L. olivaceum* species complex and is most closely related to *L. hizoidum* and *L. sagmatosporum* in phylogenetic analyses. Distinguishing between these closely related species based on morphology alone is challenging due to their considerable morphological similarities in conidial size and shape, conidiogenous apparatus structure, and recorded asexual morphs (Yin et al. 2019).

#### ﻿ Masuyamyces xishanensis

Taxon classificationAnimaliaOphiostomatalesOphiostomataceae

D. Xie, H. W. Chen & D. F. Chi sp. nov.

E4B61B90-3201-5557-862F-3DCA625A7128

856953

Fig. 12

##### Etymology.

The epithet *xishanensis* (Latin) refers to the Xi Shan village from where this taxon was first isolated.

##### Diagnosis.

*Masuyamyces xishanensis* is phylogenetically distinct from all morphologically similar species, from which it can be readily distinguished using molecular sequence data for the ITS and the beta-tubulin (*βT*) (Fig. 7, Suppl. material 12).

##### Type.

**China** • Shandong Province: Yantai City, from *H. ligniperda*, Apr. 2022, Dan Xie (***holotype***HMAS 354188, dried culture prepared from NFF1608; ex-holotype culture CGMCC3.28599 = NFF1608).

##### Description.

***Sexual morph*** not observed. ***Asexual morphs*** observed both synnematous and mononematous. ***Synnematous morph***: pesotum-like, the base transparent or light brown, (112–)158–296(–345) μm tall, including the ***conidiogenous*** apparatus, (35–)87–117(–172) μm wide. ***Conidiogenous cells*** hyaline, (5–)9–18(–22) × 1–1.5 μm. ***Conidia*** hyaline, one-celled, cylindrical, (2–)2.5–4(–5) × (1–)1.5–2(–2) μm. ***Mononematous morph***: hyalorhinocladiella-like, ***conidiogenous cells*** arising directly from mycelium, (5–)7–39(–61) × (0.5–)1–1.5(–2) µm; ***conidia*** hyaline, single-celled, smooth, obovoid to oblong, (2–)2.5–3.5(–4) × (0.5–)1–1.5(–2) μm.

##### Culture characteristics.

Colonies on 2% MEA medium slow growing in the dark, reaching 72 mm in diam. in 15 days at 25 °C, growth rate up to 4.5 mm/day at the fastest, the colonies margin smooth, part of the mycelium grows in the agar, pure white. Optimal growth temperature 30 °C, no growth observed at 5 °C, slow growth at 10 °C.

**Figure 12.**
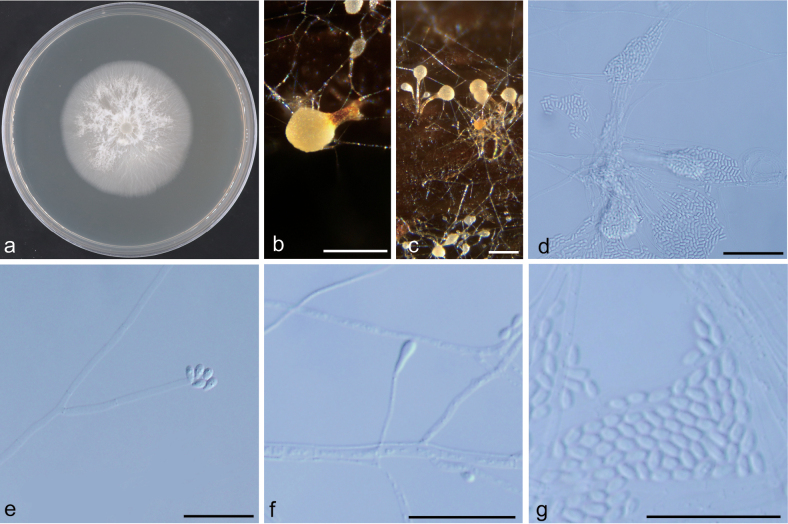
Morphological characteristics of *Masuyamyces xishanensis* sp. nov. (NFF1608). a. Fifteen-day-old culture on 2% MEA; b, c. Pesotum-like asexual morph; d. Pesotum-like asexual morph: conidiogenous cells and conidia; e–g. Hyalorhinocladiella-like asexual morph: conidiogenous cells and conidia. Scale bars: 200 μm (b, c); 20 μm (d–g).

##### Ecology.

Isolated from migratory beetles living in *Pinus* hosts. Insect vector: *H. ligniperda*.

##### Distribution.

Currently known only from Shandong Province, China.

##### Additional specimens examined.

**China** • Shandong Province: Yantai City, from *H. ligniperda*, Apr.2022, D. Xie (living culture NFF1609).

##### Notes.

*Masuymyces xishanensis* is phylogenetically most closely related to *M. dongshanensis*. Morphologically, *M. xishanensis* differs from *M. dongshanensis* by the absence of ascomatal necks, a more conspicuous pesotum-like stage, and a darker basal pigmentation. Based on both phylogenetic and morphological evidence, we propose the recognition of *M. xishanensis* as a novel species.

#### ﻿ Masuyamyces dongshanensis

Taxon classificationAnimaliaOphiostomatalesOphiostomataceae

D. Xie, H. W. Chen & D. F. Chi sp. nov.

64C276C5-75EE-587D-9C1E-83EC036549F0

856954

Fig. 13

##### Etymology.

The epithet *dongshanensis* (Latin) refers to the Dong Shan village from where this taxon was first isolated.

##### Diagnosis.

*Masuyamyces dongshanensis* is phylogenetically distinct from all morphologically similar species, from which it can be readily distinguished using molecular sequence data for the ITS and the beta-tubulin (*βT*) (Fig. 7, Suppl. material 12).

##### Type.

**China** • Shandong Province: Yantai City, from *H. ligniperda*, Apr. 2023, D. an Xie (***holotype***HMAS 354191, dried culture prepared from NFF1645; ex-holotype culture CGMCC3.28603 = NFF1645).

##### Description.

***Sexual morph*** perithecial. ***Perithecia*** appeared after 35 days of cultivation on sterilized wooden chips or 30 days of cultivation on 2%MEA, superficial or partly embedded, globose, black, the outer layer with hyphal ornamentation, (249–)367–606(–727) μm diam. ***Ascomatal*** necks black, straight or slightly curved, (410–)480–1038(–1849) µm long, (77–)107–161(–195) µm wide at base, (18–)22–41(–52) µm wide at the apex. In culture, necks sometimes 2–4 per ascoma. Asci and ascospores were not observed. ***Asexual morphs*** observed both synnematous and mononematous. ***Synnematous morph***: pesotum-like, the base transparent or light yellow, aggregating into a transparent mucilaginous spore drop, (64–)96–218(–316) μm tall, including the ***conidiogenous*** apparatus, (40–)58–108(–149) μm wide. ***Mononematous morph***: hyalorhinocladiella-like, ***conidiogenous cells*** arising directly from mycelium, (22–)23–165(–359) × (2–)2.5–3.5(–4) µm; ***conidia*** hyaline, single-celled, smooth, obovoid to bacilliform, (6–) 7–9(–10) × (2–)2.5–3 (–4) μm.

##### Culture characteristics.

Colonies on 2% MEA medium slow growing in the dark, reaching 72 mm in diam. in 15 days at 25 °C, growth rate up to 4.5 mm/day at the fastest, part of the mycelium grows in the agar, pure white. Optimal growth temperature 30 °C, slow growth at 5–10 °C.

**Figure 13.**
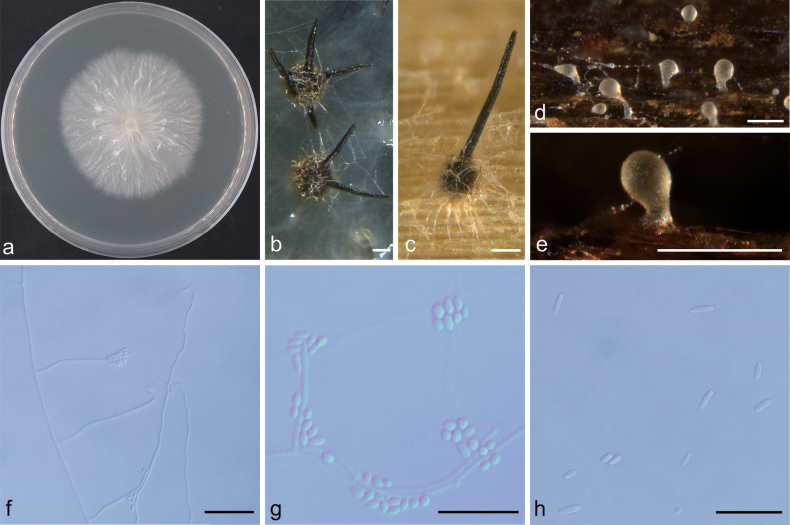
Morphological characteristics of *Masuyamyces dongshanensis* sp. nov. (NFF1645). a. Fifteen-day-old culture on 2% MEA; b, c. Perithecium; d, e. Pesotum-like asexual morph; f–h. Hyalorhinocladiella-like asexual morph: conidiogenous cells and conidia. Scale bars: 200 μm (b, c); 100 μm (d, e); 30 μm (f); 20 μm (g, h).

##### Ecology.

Isolated from migratory beetles living in *Pinus* hosts. Insect vector: *H. ligniperda*.

##### Additional specimens examined.

**China** • Shandong Province: Yantai City, from *H. ligniperda*, Apr. 2023, D. Xie (living culture NFF1646).

##### Distribution.

Currently known only from Shandong Province, China.

##### Notes.

*Masuymyces dongshanensis* is phylogenetically most closely related to *M. xishanensis*. Morphologically, it can be distinguished from *M. xishanensis* by the presence of ascomatal necks and its distinctive pesotum-like asexual morph, which is transparent, mucilaginous, and aggregates into irregularly edged, chip-like masses. Based on both phylogenetic and morphological evidence, we propose the recognition of *M. dongshanensis* as a novel species.

### ﻿Isolation and intercontinental comparison analysis of ophiostomatoid fungi from *Hylurgus ligniperda* in China

In this study, ophiostomatoid fungi were isolated from gallery-derived and trap-collected adults of *H. ligniperda*. Among these, *O. ips* exhibited the highest isolation frequency (36.55%), followed by *L. radiaticola* (14.94%) and *L. koreanum* (11.72%) (Table 3). Trap-captured adults yielded the greatest diversity of ophiostomatoid fungi (12 species), whereas gallery-derived adults showed the highest proportion of isolates (53.3%). Notably, the fungal diversity associated with gallery adults was lower (only seven species), though common ophiostomatoid species were isolated at higher frequencies compared with trap-captured adults. Known species such as *Gr. translucens*, along with new species *C. pseudoweihaiensis*, *C. pseudoyantaiensis*, *Gr. jiuguanense*, *M. xishanensis*, and *M. dongshanensis*, were isolated only from trap-captured adults. In contrast, the new species *L. ligniperdae* was only obtained from gallery adults. Ophiostomatoid fungi isolated from both gallery-derived and trap-collected adults included *H. taylorii*, *G. huntii*, *L. koreanum*, *L. radiaticola*, *M. pallidulus*, and *O. ips*. Comparative analysis of isolation frequencies between host sources revealed that *G. huntii* (*χ*² = 7.403, *p* = 0.0065), *L. koreanum* (*χ*² = 6.911, *p* = 0.0086), and *L. radiaticola* (*χ*² = 19.388, *p* < 0.0001) were isolated significantly more frequently from gallery adults than from trap-captured adults. In contrast, no significant differences in isolation frequency were observed between the gallery-derived and trap-collected adults for *H. taylorii* (*χ*² = 0.013, *p* = 0.91), *M. pallidulus* (*χ*² = 0.893, *p* = 0.345), or *O. ips* (*χ*² = 0.129, *p* = 0.719) (Suppl. material 1).

**Table 3.** Strains numbers and percentage of ophiostomatoid fungi associated with adult *Hylurgus ligniperda* in Shandong Province, China.

Genus	Taxon	Species^1^	Numbers of isolates (n = 326) ^2,3^		Total	Total isolation frequency (%)
			H. ligniperda adults in traps (isolation frequency %)	H. ligniperda adults in galleriesv (isolation frequency %)		
* Ceratocystiopsis *	1	C. pseudoweihaiensis sp. nov (NFF1617)	11(5.42)	0	11	2.53
	2	C. pseudoyantaiensis sp. nov (NFF1618)	11(5.42)	0	11	2.53
* Graphilbum *	3	*Gr. Translucens*	15(7.39)	0	15	3.45
	4	Gr. jiuguanense sp. nov (NFF1626)	9(4.43)	0	9	2.07
* Hawksworthiomyces *	5	*H.w. Taylorii*	11(5.42)	12(5.17)	23	5.29
* Leptographium *	6	*G. huntii*	9(4.43)	27(11.64^***^)	36	8.28
	7	*L. koreanum*	15(7.39)	36(15.52^***^)	51	11.72
	8	*L. radiaticola*	14(6.90)	51(21.98^***^)	65	14.94
	9	L. ligniperdae sp. nov (NFF1605)	0	9(3.88)	9	2.07
* Masuyamyces *	10	*M. pallidulus*	17(8.37)	14(6.03)	31	7.13
	11	M. xishanensis sp. nov (NFF1608)	7(3.45)	0	7	1.61
	12	M. dongshanensis sp. nov (NFF1645)	8(3.94)	0	8	1.84
* Ophiostoma *	13	*O. ips*	76(37.44)	83(35.78)	159	36.55
Total			203(46.67)	232(53.33)	435	100.00

1. Species named in black bold are novel species in this study. 2.n = The number of *H. ligniperda* adults sampled in this study. 3. The asterisk (*) indicates a significant difference in the isolation frequency of the same fungal species between adults in traps and galleries. *** indicates *p* < 0.001, no * indicates no significant difference.

**Figure 14.**
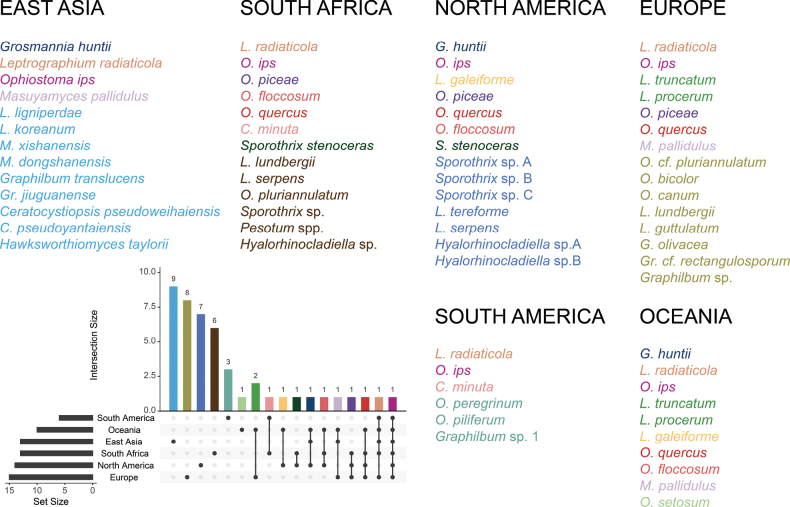
Upset diagram showing overlaps of the ophiostomatoid fungal communities associated with *Hylurgus ligniperda* in East Asia, Americas, South Africa, Europe and Oceania. The left bar graph shows the number of ophiostomatoid fungal species identified in each continent. The horizontally aligned dots on the right correspond to their respective continental regions shown on the left. Vertically, these dots connect to the bars above, representing the quantities of both endemic and shared ophiostomatoid fungal species across continents. The connecting lines represent shared fungal species. The colors of the bars match those of the fungal names in the diagram.

The current results were compared with extensive research on the global distribution of ophiostomatoid fungi associated with *H. ligniperda* across five continents (Asia, Africa, Europe, the Americas, and Oceania) (Fig. 14, Suppl. material 2, and references therein). Based on available data, 45 species have been identified in association with *H. ligniperda*. Documented records indicate that Europe exhibits the highest species diversity (15 species), followed by North America (14 species), South Africa (13 species), East Asia (13 species), Oceania (ten species), and South America (six species). Ophiostomatoid fungal communities show significant divergence among continents. Seven species associated with *H. ligniperda* exhibit transcontinental distributions (occurring on ≥ three continents). The numbers of endemic fungal species in each region are as follows: East Asia (nine species), Europe (eight species), North America (seven species), South Africa (six species), South America (three species), and Oceania (one species). *Ophiostoma ips* is the only species shared across all six regions. *Ophiostoma quercus* (Georgev.) Nannf. has been reported in North America, South Africa, Europe, and Oceania but was not isolated in the present study. Our isolation of *G. huntii* aligns with earlier records from North America and Oceania. Similarly, *M. pallidulus* identified in this study has also been documented in Europe and Oceania in association with this bark beetle.

## ﻿Discussion

Currently, *H. ligniperda* has successfully colonized and caused significant damage to coastal shelter forests in the Yanwei region of Shandong Province, China. In this study, fungi were isolated from adults *H. ligniperda* obtained from galleries and traps. A total of 425 strains of fungi associated with longhorn beetles were isolated, marking the first investigation into ophiostomatoid communities associated with *H. ligniperda* in China. Although fungal isolation is influenced by various environmental factors, including individual insect variability and environmental contamination during the isolation process, this study employed extensive sampling and isolation techniques. It is anticipated that the findings will provide a reliable representation of the fungal communities of ophiostomatoid fungi associated with *H. ligniperda* in Yantai and Weihai, Shandong Province, China. Based on the phylogenetic analysis of multigene fragments and observation of microscopic characteristics, 13 species belonging to six genera were identified. They included seven known species, viz. *Gr. translucens*, *H.w. taylorii*, *G. huntii*, *L. koreanum*, *L. radiaticola*, *M. pallidulus*, and *O. ips*. And six previously undescribed species, viz. *C. pseudoweihaiensis*, *C. pseudoyantaiensis*, *Gr. jiuguanense*, *L. ligniperdae*, *M. xishanensis*, and *M. dongshanensis*.

The ophiostomatoid fungal communities associated with gallery-derived and trap-captured *H. ligniperda* adults were significantly different. The symbiotic relationships between bark beetles and ophiostomatoid fungi appear to change with changes in the environmental conditions in which the bark beetles reside. The higher number of ophiostomatoid fungi isolated from bark beetles captured in traps may be attributed to the beetles’ exposure to a broader range of environmental fungi at this stage, rather than solely harboring specific fungi. This finding is consistent with previous studies reporting differences in species composition and isolation frequency of fungi associated with bark beetles across different countries and regions (Taerum et al. 2013; Wang et al. 2020), indicating that beetles acquire and carry environmental fungi. Furthermore, studies have shown that the fungi carried by beetles not only influence nutrient acquisition but also assist in degrading tree defensive compounds, thereby affecting the fitness of the beetles (Ayres et al. 2000; Cale et al. 2016; Six and Elser 2019; Zhao et al. 2019). The limited diversity of ophiostomatoid fungi species observed within the galleries, coupled with a high frequency of isolation, suggests that these fungi may facilitate the colonization of bark beetles in the region.

Currently, *O. ips* is distributed worldwide and associated with various beetles that infest conifers, contributing to wood bluing and, in conjunction with bark beetles, weakening trees and accelerating forest decline (Raffa and Smalley 1988; Klepzig et al. 1991; Pastirčáková et al. 2018). The fungus has also been isolated from several bark beetle-associated mites in Yunnan, China (Chang et al. 2017). There is significant interest in the relationship between *O. ips* and *Bursaphelenchus xylophilus* (Steiner & Bührer) Nickle (Niu et al. 2012; Suh et al. 2013; Zhao et al. 2014; Wang et al. 2018). In this study, *O. ips* exhibited the highest isolation rate, aligning with findings from previous research conducted on ophiostomatoid fungi associated with pines infected by *Cryphalus piceae* Ratzeburg (Chang et al. 2021), *Bursaphelenchus xylophilus* and *Monochamus alternatus* Hope (Wang et al. 2018) in Shandong, China. *Ophiostoma ips* was isolated from bark beetles collected across various countries, with the frequency of isolation varying by region (Zhou et al. 2004c; Ray et al. 2006; Davydenko et al. 2014; De Errasti et al. 2018). *Ophiostoma ips* has been reported from *H. ligniperda* across all six continents and exhibited the highest isolation frequency in the present study. This pattern may be attributed to its broad global distribution combined with its status as the most frequently isolated ophiostomatoid fungus in Shandong Province, China.

The genus *Leptographium* accommodates *L. radiaticola*, *L. koreanum*, *G. huntii*, and *L. ligniperdae*, all of which showed significantly higher isolation frequencies from gallery-derived than trap-captured adults, suggesting their specialized adaptation to the gallery environment. *Leptographium radiaticola* was the second most abundant ophiostomatoid fungus isolated in this study. Initially it was described as being isolated from *P. radiata* D. Don imported to Korea (Kim et al. 2005a), but this is not the first time the species has been spotted. *Leptographium radiaticola* has been previously identified in isolates from South Africa, Chile, Sweden, and California (Zhou et al. 2001; Zhou et al. 2004b; Kim et al. 2011). This is not the first instance of *L. radiaticola* being recorded in China. It was initially isolated from the gallery of *Dendroctonus valens* on *P. tabuliformis* in Shanxi Province, China (Lu et al. 2009). Subsequently, it was isolated again in Yunnan Province, China, during a survey of ophiostomatoid fungi affecting coniferous beetles and beetle-associated mites (Chang et al. 2017). These two reports involved different beetles that harm various conifers. This study reports the first occurrence of *L. radiaticola* in Shandong Province, China. Additionally, the sequences obtained here were distinct from those of strains previously isolated in the Yunnan region of China (Chang et al. 2017). This fungus is widely distributed globally and is particularly associated with *H. ligniperda*. The strain isolated in South Africa was obtained from *H. ligniperda* (Zhou et al. 2001), while those in Chile were isolated from *Hylastes ater* and *H. ligniperda* galleries on *P. radiata* (Zhou et al. 2004c). In California, the fungus was isolated from *H. ligniperda*, which poses a threat to *P. halepensis* Mill. and *P. pinea* L. (Kim et al. 2011). *Leptographium radiaticola* was isolated from *H. ligniperda*, an exotic bark beetle, within pine plantations in Argentina. The fungus has also been reported from *H. ligniperda* on *P. taeda* L. and *P. elliotti* Engelm. (De Errasti et al. 2018). Furthermore, during a survey of the fungal community of ophiostomatoid fungi associated with pine and pine bark beetles in southeastern Australia, this fungus was isolated from *H. ater* and *H. ligniperda*, both of which are detrimental to *P. radiata* (Trollip et al. 2021). It is likely that *L. radiaticola* from the Shandong region of China was introduced alongside *H. ligniperda*. Research has revealed that multiple beetle species harbor stable symbiotic partners (Lewinsohn et al. 1994; Six and Bentz 2003), and such bark beetle–fungus associations contribute to enhancing host adaptability by assisting the beetles in overcoming tree defense mechanisms (Six 2020). The close association between *H. ligniperda* and *L. radiaticola* may be attributed to the fungus’s ability to facilitate the colonization and development of *H. ligniperda*. This is further supported by the observation that the separation frequency within the adults’ galleries was significantly higher than that of the adults in traps in this study, suggesting that *H. ligniperda* in the gallery requires more *L. radiaticola* to withstand the host.

*Leptographium koreanum* was isolated and described from *P. densiflora* and *P. koraiensis* Siebold & Zucc. infested by *Tomicus piniperda* Linnaeus in Korea (Kim et al. 2005b). In a comprehensive survey of ophiostomatoid fungi associated with *Dendroctonus valens* in eastern North America, western North America, and China, it was observed that *L. koreanum* is predominantly confined to Asia. However, this fungus was also isolated in eastern North America, albeit at a lower prevalence compared to China (Taerum et al. 2013). Pathogenicity tests demonstrated that *L. koreanum* could induce significant necrosis in the bark of *P. tabuliformis* (Lu et al. 2009). Following its invasion of the Yanwei region of China, *H. ligniperda* established contact with *L. koreanum*. Numerous invasive beetles establish novel associations with indigenous fungi at new invasion sites (Jacobs et al. 2003; Hausner et al. 2005), leading to the hypothesis that *L. koreanum* may facilitate the colonization of *H. ligniperda*. *Grosmannia huntii*, a group of fungi well-suited for transmission by bark beetles, exhibits a broad geographical distribution, encompassing regions such as Chile, New Zealand, Argentina, North America, and Australia(Zhou et al. 2004c; Reay et al. 2005; Ray et al. 2006; Taerum et al. 2013; De Errasti et al. 2017; Trollip et al. 2021). Notably, *H. ligniperda* infestations have been documented in these regions, although *G. huntii* has not yet been reported in China. The strains isolated in this study displayed high similarity to Australian strains obtained from *H. Ater* and *H. ligniperda* (Trollip et al. 2021). It is postulated that *G. huntii* is closely associated with *H. ligniperda* and may have been introduced to China alongside *H. ligniperda*, where it has successfully colonized. *Leptographium ligniperdae* is a member of the *L. olivaceum* species complex (De Beer et al. 2022). In this study, it was exclusively isolated from adult *H. ligniperda* within their galleries. Multigene phylogenetic analysis revealed that it is closely related to *L. rhizoidum* and *L. sagmatosporum*. *Leptographium rhizoidum* was isolated and characterized from *H. ater* and *H. attenuatus* Erichson, which infest *P. radiata* in Spain. Currently known insect vectors include *H. ater*, *H. attenuatus*, *Hylurgops palliatus* Gyllenhal, and *Ips sexdentatus* Börner (Yin et al. 2019). *Leptographium sagmatosporum* was initially isolated from bark beetle galleries and fresh wood surfaces on *Picea mariana* (Mill.) Britton, Sterns & Poggenb., *P. resinosa* Aiton, and *P. strobus* L. (Yin et al. 2019). The discovery of *L. ligniperdae* not only contributes to the diversity of the *L. olivaceum* species complex but also provides valuable insights into the ophiostomatoid fungal community associated with *H. ligniperda*.

Species of *Masuyamyces* isolated in this study include *M. pallidulus* and two novel species, *M. xishanensis* and *M. dongshanensis* (De Beer et al. 2022). In Finland, *M. pallidulus* was initially isolated from *P. sylvestris*, which is associated with various beetle species (Linnakoski et al. 2010). In Poland, *M. pallidulus* was isolated from the roots of dead trees and subsequently identified during a survey of ophiostomatoid fungi linked to root-eating beetles on *P. sylvestris* in the region (Jankowiak and Bilański 2013). *Masuyamyces pallidulus* is also present in Australia, including isolates from *H. ligniperda* (Trollip et al. 2021). In the genus *Hawksworthiomyces*, *H.w. taylorii* was first isolated from *Eucalyptus* poles within the soil layer in South Africa, with an optimal growth temperature of 30 °C (De Beer et al. 2016b). This fungus had not been previously isolated from *H. ligniperda*. Given that *H. ligniperda* primarily feeds on the roots of its host and interacts with the soil environment, it is hypothesized that the fungus is a soil-associated organism.

Certain fungi belonging to *Ceratocystiopsis* and *Graphilbum* were exclusively isolated from adults of *H. ligniperda* in traps. In the genus *Ceratocystiopsis*, *C. pseudoweihaiensis* is closely related to *C. weihaiensis*, while *C. pseudoyantaiensis* is closely related to *C. yantaiensis*. Both *C. weihaiensis* and *C. yantaiensis* have been isolated and reported from the *Cryphalus piceae* gallery on *P. thunbergii* in Shandong Province, China (Chang et al. 2021). Within the genus *Graphilbum*, *Gr. translucens*, and *Gr. jiuguanense* are identified. Multilocus phylogenetic analysis indicated that *Gr. jiuguanense* formed a distinct lineage closely related to *Gr. niveum*. Furthermore, both *Gr. translucens* and *Gr. niveum* were isolated from *Cryphalus piceae* galleries on *P. densiflora* and *P. thunbergii* in Shandong Province, China (Chang et al. 2021). This aligns with the ecological niche of *H. ligniperda* in China, suggesting that *H. ligniperda* acquires native fungi from the environment, although some fungi may undergo changes as they adapt to new host insects. The absence of these fungi in adult samples within the gallery may be attributed to their weak association with *H. ligniperda*.

The significant divergence in ophiostomatoid fungal communities observed across continents demonstrates that regional variation serves as the primary driver of fungal community differentiation in *H. ligniperda* associations. In this study, six novel fungal species along with several long-term fungal partners were isolated from *H. ligniperda* adults, indicating that the beetle develops specialized fungal assemblages adapted to local environments across different regions to facilitate its colonization and expansion into new habitats.

## ﻿Conclusions

Our findings revealed a fungal community of ophiostomatoid fungi associated with *H. ligniperda*, which has successfully colonization in Yanwei, Shandong Province, China. We identified a total of 13 species across six genera, including six novel species. *Ophiostoma ips* and *L. radiaticola* emerged as the dominant species, with isolation frequencies of 36.55% and 14.94%, respectively. Variations in the ophiostomatoid communities associated with *H. ligniperda* across different regions may be attributed to environmental differences. Nevertheless, the presence of certain fungi, such as *O. ips*, *L. radiaticola*, *G. huntii*, and *M. pallidulus*, remains consistent. Notably, the *L. radiaticola* and *G. huntii* strains isolated in this study clustered into a single clade with high sequence similarity to strains from southeast Australia, also isolated from *H. ligniperda*. It is hypothesized that the source population of *H. ligniperda* established in Shandong, China, is the same as that of the Australian population. Furthermore, native fungi and newly identified species closely related to native fungi were isolated from *H. ligniperda*, suggesting that insects in newly invaded areas establish interactions with native fungi, which in turn adapt to these insects through evolutionary changes. Understanding this novel association formed by *H. ligniperda* in the Yantai and Weihai regions of China is crucial for future management strategies of this insect. The successful colonization of alien invasive insects in new environments is influenced by numerous factors, among which the fungi associated with bark beetles play a significant role. Consequently, it is imperative to comprehend the relationship between bark beetles and their associated fungi, as this knowledge will facilitate timely control of alien invasive insect populations and help prevent substantial economic losses.

## Supplementary Material

XML Treatment for Ceratocystiopsis pseudoweihaiensis

XML Treatment for Ceratocystiopsis pseudoyantaiensis

XML Treatment for Graphilbum jiuguanense

XML Treatment for Leptographium ligniperdae

XML Treatment for Masuyamyces xishanensis

XML Treatment for Masuyamyces dongshanensis

## ﻿Additional information

### Use of AI

No use of AI was reported.

### Adherence to national and international regulations

All the fungal strains used in this study have been legally obtained, respecting the Convention on Biological Diversity (Rio Convention).

### Author ORCIDs

Dan Xie https://orcid.org/0000-0002-7454-1657

Huanwen Chen https://orcid.org/0000-0002-9423-5120

Niya Jia https://orcid.org/0000-0002-0281-3924

Fang Niu https://orcid.org/0000-0001-5262-6863

Xiaomei Wang https://orcid.org/0000-0001-8857-4663

Jia Yu https://orcid.org/0000-0001-5735-6217

Defu Chi https://orcid.org/0000-0003-1366-7560

### Data availability

All sequences generated during this study have been submitted to GenBank.

## Supplementary materials

Supplementary material 1 Comparative analysis of isolation frequency differences of fungi obtained from both gallery-derived and trap-collected adults Data type docx Explanation note Nucleotide substitution models obtained from ModelFinder v2.2.0. https://binary.pensoft.net/file/1452559This dataset is made available under the Open Database License (http://opendatacommons.org/licenses/odbl/1.0/). The Open Database License (ODbL) is a license agreement intended to allow users to freely share, modify, and use this Dataset while maintaining this same freedom for others, provided that the original source and author(s) are credited.Author: Dan Xie

Supplementary material 2 Comparison of ophiostomatoid associates of *Hylurgus ligniperda* in East Asia, Americas, South Africa, Europe and Oceania Data type docx Explanation note Comparison of ophiostomatoid associates of *Hylurgus ligniperda* in East Asia, Americas, South Africa, Europe and Oceania. https://binary.pensoft.net/file/1452560This dataset is made available under the Open Database License (http://opendatacommons.org/licenses/odbl/1.0/). The Open Database License (ODbL) is a license agreement intended to allow users to freely share, modify, and use this Dataset while maintaining this same freedom for others, provided that the original source and author(s) are credited.Author: Dan Xie

Supplementary material 3 ML tree of *Ceratocystiopsis* generated from the ITS sequence data Data type png Explanation note ML tree of *Ceratocystiopsis* generated from the ITS sequence data. Sequences generated in this study are printed in bold. Bold branches indicate posterior probability values ≥ 0.9, while ML SH-aLRT/UFBooT values of ≥ 75% are recorded at nodes, < 75% are indicated by the symbol *. T = ex-type cultures. https://binary.pensoft.net/file/1452561This dataset is made available under the Open Database License (http://opendatacommons.org/licenses/odbl/1.0/). The Open Database License (ODbL) is a license agreement intended to allow users to freely share, modify, and use this Dataset while maintaining this same freedom for others, provided that the original source and author(s) are credited.Author: Dan Xie

Supplementary material 4 ML tree of *Ceratocystiopsis* generated from the *βT* sequence data Data type png Explanation note ML tree of *Ceratocystiopsis* generated from the *βT* sequence data. Sequences generated in this study are printed in bold. Bold branches indicate posterior probability values ≥ 0.9, while ML SH-aLRT/UFBooT values of ≥ 75% are recorded at nodes, < 75% are indicated by the symbol *. T = ex-type cultures. https://binary.pensoft.net/file/1452562This dataset is made available under the Open Database License (http://opendatacommons.org/licenses/odbl/1.0/). The Open Database License (ODbL) is a license agreement intended to allow users to freely share, modify, and use this Dataset while maintaining this same freedom for others, provided that the original source and author(s) are credited.Author: Dan Xie

Supplementary material 5 ML tree of *Ceratocystiopsis* generated from the *TEF1-α* sequence data Data type png Explanation note ML tree of *Ceratocystiopsis* generated from the *TEF1-α* sequence data. Sequences generated in this study are printed in bold. Bold branches indicate posterior probability values ≥ 0.9, while ML SH-aLRT/UFBooT values of ≥ 75% are recorded at nodes, < 75% are indicated by the symbol *. T = ex-type cultures. https://binary.pensoft.net/file/1452563This dataset is made available under the Open Database License (http://opendatacommons.org/licenses/odbl/1.0/). The Open Database License (ODbL) is a license agreement intended to allow users to freely share, modify, and use this Dataset while maintaining this same freedom for others, provided that the original source and author(s) are credited.Author: Dan Xie

Supplementary material 6 ML tree of *Graphilbum* generated from the ITS sequence data Data type png Explanation note ML tree of *Graphilbum* generated from the ITS sequence data. Sequences generated in this study are printed in bold. Bold branches indicate posterior probability values ≥ 0.9, while ML SH-aLRT/UFBooT values of ≥ 75% are recorded at nodes, < 75% are indicated by the symbol *. T = ex-type cultures https://binary.pensoft.net/file/1452564This dataset is made available under the Open Database License (http://opendatacommons.org/licenses/odbl/1.0/). The Open Database License (ODbL) is a license agreement intended to allow users to freely share, modify, and use this Dataset while maintaining this same freedom for others, provided that the original source and author(s) are credited.Author: Dan Xie

Supplementary material 7 ML tree of *Graphilbum* generated from the *TEF1-α* sequence data Data type png Explanation note ML tree of *Graphilbum* generated from the *TEF1-α* sequence data. Sequences generated in this study are printed in bold. Bold branches indicate posterior probability values ≥ 0.9, while ML SH-aLRT/UFBooT values of ≥ 75% are recorded at nodes, < 75% are indicated by the symbol *. T = ex-type cultures. https://binary.pensoft.net/file/1452565This dataset is made available under the Open Database License (http://opendatacommons.org/licenses/odbl/1.0/). The Open Database License (ODbL) is a license agreement intended to allow users to freely share, modify, and use this Dataset while maintaining this same freedom for others, provided that the original source and author(s) are credited.Author: Dan Xie

Supplementary material 8 ML tree of *Graphilbum* generated from the *CAL* sequence data Data type png Explanation note ML tree of *Graphilbum* generated from the *CAL* sequence data. Sequences generated in this study are printed in bold. Bold branches indicate posterior probability values ≥ 0.9, while ML SH-aLRT/UFBooT values of ≥ 75% are recorded at nodes, < 75% are indicated by the symbol *. T = ex-type cultures. https://binary.pensoft.net/file/1452566This dataset is made available under the Open Database License (http://opendatacommons.org/licenses/odbl/1.0/). The Open Database License (ODbL) is a license agreement intended to allow users to freely share, modify, and use this Dataset while maintaining this same freedom for others, provided that the original source and author(s) are credited.Author: Dan Xie

Supplementary material 9 ML tree of *L. olivaceum* complex generated from the *βT* sequence data Data type png Explanation note ML tree of *L. olivaceum* complex generated from the *βT* sequence data. Sequences generated in this study are printed in bold. Bold branches indicate posterior probability values ≥ 0.9, while ML SH-aLRT/UFBooT values of ≥ 75% are recorded at nodes, < 75% are indicated by the symbol *. T = ex-type cultures. https://binary.pensoft.net/file/1452567This dataset is made available under the Open Database License (http://opendatacommons.org/licenses/odbl/1.0/). The Open Database License (ODbL) is a license agreement intended to allow users to freely share, modify, and use this Dataset while maintaining this same freedom for others, provided that the original source and author(s) are credited.Author: Dan Xie

Supplementary material 10 ML tree of *L. olivaceum* complex generated from the *TEF1-α* sequence data Data type png Explanation note ML tree of *L. olivaceum* complex generated from the *TEF1-α* sequence data. Sequences generated in this study are printed in bold. Bold branches indicate posterior probability values ≥ 0.9, while ML SH-aLRT/UFBooT values of ≥ 75% are recorded at nodes, < 75% are indicated by the symbol *. T = ex-type cultures. https://binary.pensoft.net/file/1452568This dataset is made available under the Open Database License (http://opendatacommons.org/licenses/odbl/1.0/). The Open Database License (ODbL) is a license agreement intended to allow users to freely share, modify, and use this Dataset while maintaining this same freedom for others, provided that the original source and author(s) are credited.Author: Dan Xie

Supplementary material 11 ML tree of *L. olivaceum* complex generated from the *CAL* sequence data Data type png Explanation note ML tree of *L. olivaceum* complex generated from the *CAL* sequence data. Sequences generated in this study are printed in bold. Bold branches indicate posterior probability values ≥ 0.9, while ML SH-aLRT/UFBooT values of ≥ 75% are recorded at nodes, < 75% are indicated by the symbol *. T = ex-type cultures. https://binary.pensoft.net/file/1452569This dataset is made available under the Open Database License (http://opendatacommons.org/licenses/odbl/1.0/). The Open Database License (ODbL) is a license agreement intended to allow users to freely share, modify, and use this Dataset while maintaining this same freedom for others, provided that the original source and author(s) are credited.Author: Dan Xie

Supplementary material 12 ML tree of *Masuyamyces* and *Ophiostoma ips* complex generated from the *βT* sequence data Data type png Explanation note ML tree of *Masuyamyces* and *Ophiostoma ips* complex generated from the *βT* sequence data. Sequences generated in this study are printed in bold. Bold branches indicate posterior probability values ≥ 0.9, while ML SH-aLRT/UFBooT values of ≥ 75% are recorded at nodes, < 75% are indicated by the symbol *. T = ex-type cultures https://binary.pensoft.net/file/1452570This dataset is made available under the Open Database License (http://opendatacommons.org/licenses/odbl/1.0/). The Open Database License (ODbL) is a license agreement intended to allow users to freely share, modify, and use this Dataset while maintaining this same freedom for others, provided that the original source and author(s) are credited.Author: Dan Xie

Supplementary material 13 ML phylogeny of LSU region for isolates residing in *Leptographium*, *Ceratocystiopsis* and *Hawksworthiomyces* Data type nexus Explanation note ML phylogeny of LSU region for isolates residing in *Leptographium*, *Ceratocystiopsis* and *Hawksworthiomyces*. https://binary.pensoft.net/file/1452571This dataset is made available under the Open Database License (http://opendatacommons.org/licenses/odbl/1.0/). The Open Database License (ODbL) is a license agreement intended to allow users to freely share, modify, and use this Dataset while maintaining this same freedom for others, provided that the original source and author(s) are credited.Author: Dan Xie

Supplementary material 14 ML phylogeny of ITS region for isolates residing in *Masuyamyces*, *Ophiostoma* and *Graphilbum* Data type nexus Explanation note ML phylogeny of ITS region for isolates residing in *Masuyamyces*, *Ophiostoma* and *Graphilbum*. https://binary.pensoft.net/file/1452572This dataset is made available under the Open Database License (http://opendatacommons.org/licenses/odbl/1.0/). The Open Database License (ODbL) is a license agreement intended to allow users to freely share, modify, and use this Dataset while maintaining this same freedom for others, provided that the original source and author(s) are credited.Author: Dan Xie

Supplementary material 15 ML tree of *Ceratocystiopsis* generated from the combined (ITS + *βT* + *TEF1-α*) sequence data Data type nexus Explanation note ML tree of *Ceratocystiopsis* generated from the combined (ITS + *βT* + *TEF1-α*) sequence data. https://binary.pensoft.net/file/1452573This dataset is made available under the Open Database License (http://opendatacommons.org/licenses/odbl/1.0/). The Open Database License (ODbL) is a license agreement intended to allow users to freely share, modify, and use this Dataset while maintaining this same freedom for others, provided that the original source and author(s) are credited.Author: Dan Xie

Supplementary material 16 ML tree of *Graphilbum* generated from the combined (ITS + *TEF1-α* + *CAL*) sequence data Data type nexus Explanation note ML tree of *Graphilbum* generated from the combined (ITS + *TEF1-α* + *CAL*) sequence data. https://binary.pensoft.net/file/1452574This dataset is made available under the Open Database License (http://opendatacommons.org/licenses/odbl/1.0/). The Open Database License (ODbL) is a license agreement intended to allow users to freely share, modify, and use this Dataset while maintaining this same freedom for others, provided that the original source and author(s) are credited.Author: Dan Xie

Supplementary material 17 ML tree of *Hawksworthiomyces* generated from the combined (ITS + *TEF1-α* + *RPB2*) sequence data Data type nexus Explanation note ML tree of *Hawksworthiomyces* generated from the combined (ITS + *TEF1-α* + *RPB2*) sequence data. https://binary.pensoft.net/file/1452575This dataset is made available under the Open Database License (http://opendatacommons.org/licenses/odbl/1.0/). The Open Database License (ODbL) is a license agreement intended to allow users to freely share, modify, and use this Dataset while maintaining this same freedom for others, provided that the original source and author(s) are credited.Author: Dan Xie

Supplementary material 18 ML tree of *Leptographium* generated from the combined (*βT* + *TEF1-α*) sequence data Data type nexus Explanation note ML tree of *Leptographium* generated from the combined (*βT* + *TEF1-α*) sequence data. https://binary.pensoft.net/file/1452576This dataset is made available under the Open Database License (http://opendatacommons.org/licenses/odbl/1.0/). The Open Database License (ODbL) is a license agreement intended to allow users to freely share, modify, and use this Dataset while maintaining this same freedom for others, provided that the original source and author(s) are credited.Author: Dan Xie

Supplementary material 19 ML tree of *Masuyamyces* and *Ophiostoma ips* complex generated from the ITS sequence data Data type nexus Explanation note ML tree of *Masuyamyces* and *Ophiostoma ips* complex generated from the ITS sequence data. https://binary.pensoft.net/file/1452577This dataset is made available under the Open Database License (http://opendatacommons.org/licenses/odbl/1.0/). The Open Database License (ODbL) is a license agreement intended to allow users to freely share, modify, and use this Dataset while maintaining this same freedom for others, provided that the original source and author(s) are credited.Author: Dan Xie

Supplementary material 20 ML tree of *Ceratocystiopsis* generated from the ITS sequence data Data type nexus Explanation note ML tree of *Ceratocystiopsis* generated from the ITS sequence data. https://binary.pensoft.net/file/1452578This dataset is made available under the Open Database License (http://opendatacommons.org/licenses/odbl/1.0/). The Open Database License (ODbL) is a license agreement intended to allow users to freely share, modify, and use this Dataset while maintaining this same freedom for others, provided that the original source and author(s) are credited.Author: Dan Xie

Supplementary material 21 ML tree of *Ceratocystiopsis* generated from the *βT* sequence data Data type nexus Explanation note ML tree of *Ceratocystiopsis* generated from the *βT* sequence data. https://binary.pensoft.net/file/1452579This dataset is made available under the Open Database License (http://opendatacommons.org/licenses/odbl/1.0/). The Open Database License (ODbL) is a license agreement intended to allow users to freely share, modify, and use this Dataset while maintaining this same freedom for others, provided that the original source and author(s) are credited.Author: Dan Xie

Supplementary material 22 ML tree of *Ceratocystiopsis* generated from the *TEF1-α* sequence data Data type nexus Explanation note ML tree of *Ceratocystiopsis* generated from the *TEF1-α* sequence data. https://binary.pensoft.net/file/1452580This dataset is made available under the Open Database License (http://opendatacommons.org/licenses/odbl/1.0/). The Open Database License (ODbL) is a license agreement intended to allow users to freely share, modify, and use this Dataset while maintaining this same freedom for others, provided that the original source and author(s) are credited.Author: Dan Xie

Supplementary material 23 ML tree of *Graphilbum* generated from the ITS sequence data Data type nexus Explanation note ML tree of *Graphilbum* generated from the ITS sequence data. https://binary.pensoft.net/file/1452581This dataset is made available under the Open Database License (http://opendatacommons.org/licenses/odbl/1.0/). The Open Database License (ODbL) is a license agreement intended to allow users to freely share, modify, and use this Dataset while maintaining this same freedom for others, provided that the original source and author(s) are credited.Author: Dan Xie

Supplementary material 24 ML tree of *Graphilbum* generated from the *TEF1-α* sequence data Data type nexus Explanation note ML tree of *Graphilbum* generated from the *TEF1-α* sequence data. https://binary.pensoft.net/file/1452582This dataset is made available under the Open Database License (http://opendatacommons.org/licenses/odbl/1.0/). The Open Database License (ODbL) is a license agreement intended to allow users to freely share, modify, and use this Dataset while maintaining this same freedom for others, provided that the original source and author(s) are credited.Author: Dan Xie

Supplementary material 25 ML tree of *Graphilbum* generated from the *CAL* sequence data Data type nexus Explanation note ML tree of *Graphilbum* generated from the *CAL* sequence data. https://binary.pensoft.net/file/1452583This dataset is made available under the Open Database License (http://opendatacommons.org/licenses/odbl/1.0/). The Open Database License (ODbL) is a license agreement intended to allow users to freely share, modify, and use this Dataset while maintaining this same freedom for others, provided that the original source and author(s) are credited.Author: Dan Xie

Supplementary material 26 ML tree of *L. olivaceum* complex generated from the *βT* sequence data Data type nexus Explanation note ML tree of *L. olivaceum* complex generated from the *βT* sequence data. https://binary.pensoft.net/file/1452584This dataset is made available under the Open Database License (http://opendatacommons.org/licenses/odbl/1.0/). The Open Database License (ODbL) is a license agreement intended to allow users to freely share, modify, and use this Dataset while maintaining this same freedom for others, provided that the original source and author(s) are credited.Author: Dan Xie

Supplementary material 27 ML tree of *L. olivaceum* complex generated from the *TEF1-α* sequence data Data type nexus Explanation note ML tree of *L. olivaceum* complex generated from the *TEF1-α* sequence data. https://binary.pensoft.net/file/1452585This dataset is made available under the Open Database License (http://opendatacommons.org/licenses/odbl/1.0/). The Open Database License (ODbL) is a license agreement intended to allow users to freely share, modify, and use this Dataset while maintaining this same freedom for others, provided that the original source and author(s) are credited.Author: Dan Xie

Supplementary material 28 ML tree of *L. olivaceum* complex generated from the *CAL* sequence data Data type nexus Explanation note ML tree of *L. olivaceum* complex generated from the *CAL* sequence data. https://binary.pensoft.net/file/1452586This dataset is made available under the Open Database License (http://opendatacommons.org/licenses/odbl/1.0/). The Open Database License (ODbL) is a license agreement intended to allow users to freely share, modify, and use this Dataset while maintaining this same freedom for others, provided that the original source and author(s) are credited.Author: Dan Xie

Supplementary material 29 ML tree of *Masuyamyces* and *Ophiostoma ips* complex generated from the *βT* sequence data Data type nexus Explanation note ML tree of *Masuyamyces* and *Ophiostoma ips* complex generated from the *βT* sequence data. https://binary.pensoft.net/file/1452587This dataset is made available under the Open Database License (http://opendatacommons.org/licenses/odbl/1.0/). The Open Database License (ODbL) is a license agreement intended to allow users to freely share, modify, and use this Dataset while maintaining this same freedom for others, provided that the original source and author(s) are credited.Author: Dan Xie
