# Supplementary material for: ﻿Ophiostomatoid fungi associated with Hylurgus ligniperda, including six new species from eastern China
Source: IMA Fungus. 2025 Oct 28;16:e169382. doi: 10.3897/imafungus.16.169382 (PMC12587175; doi:10.3897/imafungus.16.169382)
Supplement: Supplementary material 1 — Comparative analysis of isolation frequency differences of fungi obtained from both gallery-derived and trap-collected adults [file imafungus-16-e169382-s001.docx]

Additional file 1: Table S1 Comparative analysis of isolation frequency differences of fungi obtained from both gallery-derived and trap-collected adults

| **Species** | *χ*2 | *P* |
| --- | --- | --- |
| *Hawksworthiomyces taylorii* | 0.013 | 0.91 |
| *Grosmannia huntii* | 7.403 | 0.0065 |
| *Leptographium koreanum* | 6.911 | 0.0086 |
| *L. radiaticola* | 19.388 | <0.0001 |
| *Masuyamyces pallidulus* | 0.893 | 0.345 |
| *Ophiostoma ips* | 0.129 | 0.719 |
