# Supplementary material for: ﻿Ophiostomatoid fungi associated with Hylurgus ligniperda, including six new species from eastern China
Source: IMA Fungus. 2025 Oct 28;16:e169382. doi: 10.3897/imafungus.16.169382 (PMC12587175; doi:10.3897/imafungus.16.169382)
Supplement: Supplementary material 2 — Comparison of ophiostomatoid associates of Hylurgus ligniperda in East Asia, Americas, South Africa, Europe and Oceania [file imafungus-16-e169382-s002.docx]

Table S2 Comparison of ophiostomatoid associates of *Hylurgus ligniperda* in East Asia, Americas, South Africa, Europe and Oceania

| Fungal species | Genus | East Asia | North America | South America | South Africa | Europe | Oceania |
| --- | --- | --- | --- | --- | --- | --- | --- |
| ***Ophiostomatales*** |  |  |  |  |  |  |  |
| *Grosmannia huntii* | *Leptographium* spp. | this study | (Kim et al. 2011) |  |  |  | (Ray et al. 2006; Trollip et al. 2021) |
| *G. olivacea* |  |  |  |  |  | (Jankowiak and Bilański 2013) |  |
| *Leptographium koreanum* |  | this study |  |  |  |  |  |
| *L. radiaticola*^1^ |  | this study |  | (Zhou et al. 2004b; Zhou et al. 2004c; Linnakoski et al. 2012; de Errasti et al. 2017) | (Zhou et al. 2001; Zhou et al. 2004b; Zhou et al. 2006; Linnakoski et al. 2012) | (Jankowiak and Bilański 2013) | (Trollip et al. 2021) |
| *L. galeiforme* |  |  | (Kim et al. 2011) |  |  |  | (Ray et al. 2006) |
| *L. ligniperdae* |  | this study |  |  |  |  |  |
| *L. tereforme* |  |  | (Kim et al. 2011) |  |  |  |  |
| *L. serpens* |  |  | (Kim et al. 2011) |  |  |  |  |
| *L. lundberg2* |  |  |  |  | (Zhou et al. 2001) | (Jankowiak et al. 2012; Jankowiak and Bilański 2013) |  |
| *L. serpens* |  |  |  |  | (Zhou et al. 2001) |  |  |
| *L. guttulatum* |  |  |  |  |  | (Rornn et al. 2007) |  |
| *L. truncatum* |  |  |  |  |  | (Jankowiak and Bilański 2013) | (Ray et al. 2006) |
| *L. procerum* |  |  |  |  |  | (Jankowiak and Bilański 2013) | (Ray et al. 2006) |
| *Ophiostoma ips* | *Ophiostoma* spp. | this study | (Kim et al. 2011) | (Zhou et al. 2004a; Zhou et al. 2004c; de Errasti et al. 2017) | (Zhou et al. 2001; Zhou et al. 2004a) | (DAVYDENKO et al. 2014) | (Ray et al. 2006) |
| *O. piceae* |  |  | (Kim et al. 2011) |  | (Zhou et al. 2001; Zhou et al. 2006) | (Jankowiak and Bilański 2013; DAVYDENKO et al. 2014) |  |
| *O. quercus* |  |  | (Kim et al. 2011) |  | (Zhou et al. 2006) | (Jankowiak and Bilański 2013) | (Ray et al. 2006) |
| *O. floccosum* |  |  | (Kim et al. 2011) |  | (Zhou et al. 2006) |  | (Ray et al. 2006) |
| *O. peregrinum* |  |  |  | (de Errasti et al. 2017) |  |  |  |
| *O. piliferum* |  |  |  | (de Errasti et al. 2017) |  |  |  |
| *O. bicolor* |  |  |  |  |  | (DAVYDENKO et al. 2014) |  |
| *O. canum* |  |  |  |  |  | (DAVYDENKO et al. 2014) |  |
| *O. pluriannulatum* |  |  |  |  | (Zhou et al. 2001; Zhou et al. 2006) |  |  |
| *O. cf. pluriannulatum* |  |  |  |  |  | (Jankowiak and Bilański 2013) |  |
| *O. rectangulosporium* |  |  |  |  |  | (DAVYDENKO et al. 2014) |  |
| *O. setosum* |  |  |  |  |  |  | (Ray et al. 2006) |
| *Masuyamyces pallidulus* | *Masuyamyces* spp. | this study |  |  |  | (Jankowiak and Bilański 2013) | (Trollip et al. 2021) |
| *M. xishanensis* |  | this study |  |  |  |  |  |
| *M. dongshanensis* |  | this study |  |  |  |  |  |
| *Graphilbum translucens* | *Graphilbum* spp. | this study |  |  |  |  |  |
| *Gr. jiuguanense* |  | this study |  |  |  |  |  |
| *Graphilbum* sp. 1 |  |  |  | (de Errasti et al. 2017) |  |  |  |
| *Ceratocystiopsis minuta* |  |  |  | (Zhou et al. 2004c) | (Zhou et al. 2001; Zhou et al. 2006) |  |  |
| *C. pseudoweihaiensis* |  | this study |  |  |  |  |  |
| *C. pseudoyantaiensis* | *Ceratocystiopsis* spp.  *Hawksworthiomyces* spp. | this study |  |  |  |  |  |
| *Hawksworthiomyces taylorii* |  | this study |  |  |  |  |  |
| *Sporothrix stenoceras*^2^ | *Sporothrix* spp. |  | (Kim et al. 2011) |  | (Zhou et al. 2001) |  |  |
| *Sporothrix* sp. A |  |  | (Kim et al. 2011) |  |  |  |  |
| *Sporothrix* sp. B |  |  | (Kim et al. 2011) |  |  |  |  |
| *Sporothrix* sp. C |  |  | (Kim et al. 2011) |  |  |  |  |
| *Sporothrix* sp. |  |  |  |  | (Zhou et al. 2001; Zhou et al. 2006) |  |  |
| *Hyalorhinocladiella* sp. A | unknown |  | (Kim et al. 2011) |  |  |  |  |
| *Hyalorhinocladiella* sp. B |  |  | (Kim et al. 2011) |  |  |  |  |
| *Hyalorhinocladiella* sp. |  |  |  |  | (Zhou et al. 2001; Zhou et al. 2006) |  |  |
| *Pesotum* spp. |  |  |  |  | (Zhou et al. 2001; Zhou et al. 2006) |  |  |
| ***Microascales*** |  |  |  |  |  |  |  |
| *Graphium* sp. | *Graphium* spp. |  |  |  |  | (DAVYDENKO et al. 2014) |  |

1. This species previously considered as *Grosmannia. galeiformis* in original paper. The species confirmed to be *G. radiaticola* (DAVYDENKO et al. 2014). The species was subsequently reclassified as *Leptographium radiaticola* (De Beer et al. 2022).

2. This species previously considered as *Ophiostoma stenoceras* in original paper. The species was subsequently reclassified as *Sporothrix stenoceras* (De Beer et al. 2016).
