# Supplementary figures and images for: ﻿Ophiostomatoid fungi associated with Hylurgus ligniperda, including six new species from eastern China
Source: IMA Fungus. 2025 Oct 28;16:e169382. doi: 10.3897/imafungus.16.169382 (PMC12587175; doi:10.3897/imafungus.16.169382)

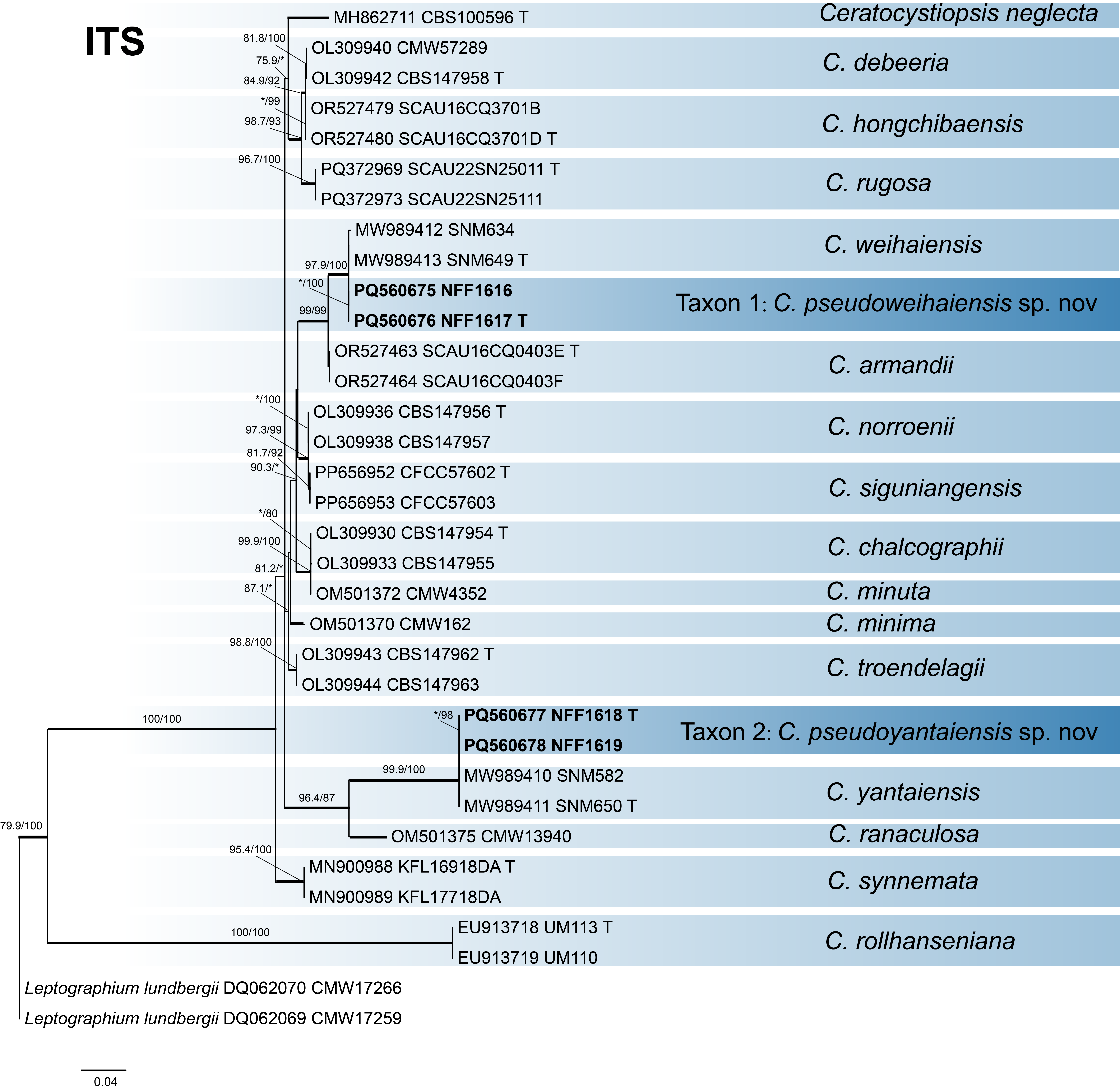

Supplement: Supplementary material 3 — ML tree of Ceratocystiopsis generated from the ITS sequence data [file imafungus-16-e169382-s003.png]

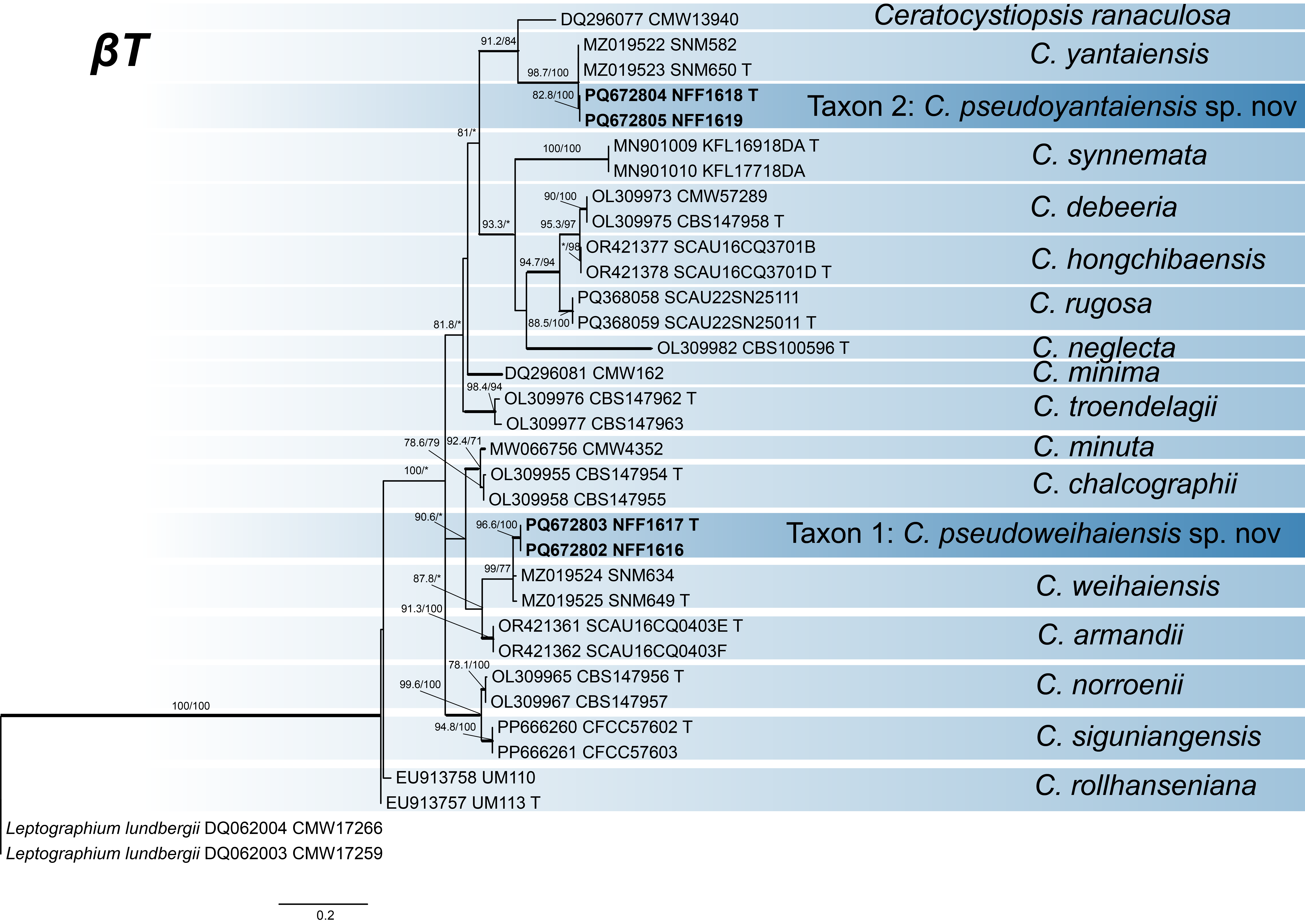

Supplement: Supplementary material 4 — ML tree of Ceratocystiopsis generated from the βT sequence data [file imafungus-16-e169382-s004.png]

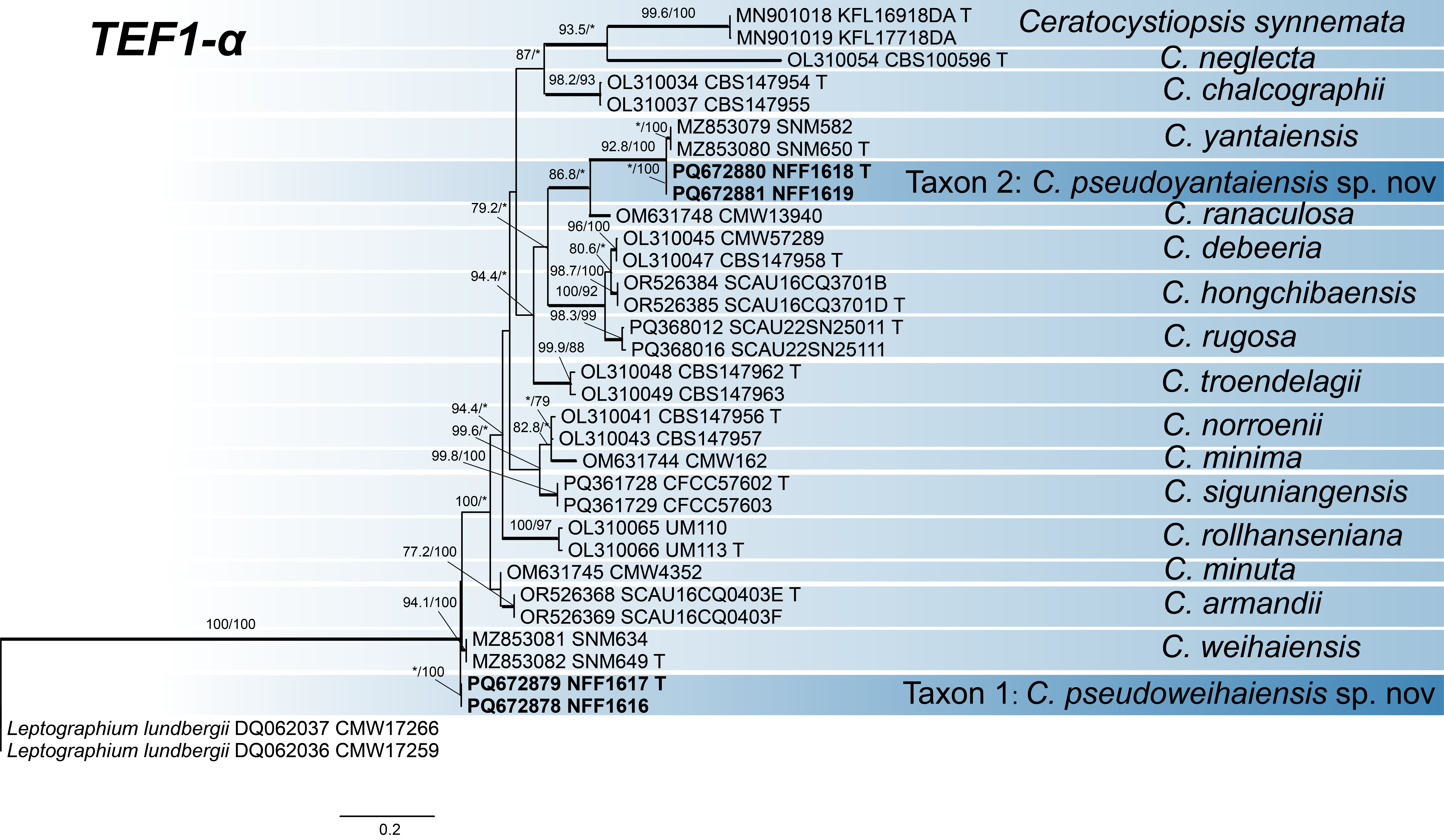

Supplement: Supplementary material 5 — ML tree of Ceratocystiopsis generated from the TEF1-α sequence data [file imafungus-16-e169382-s005.png]

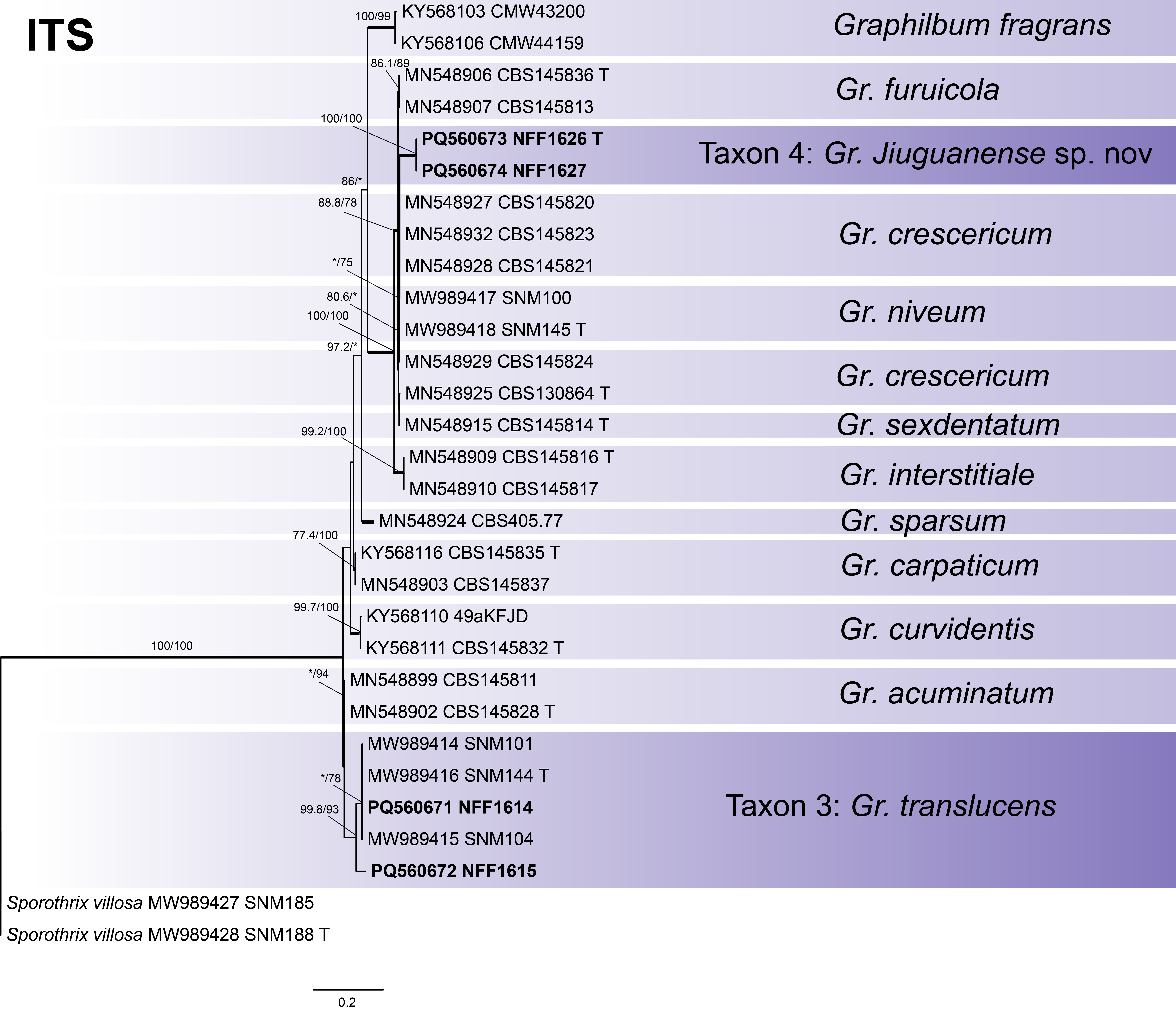

Supplement: Supplementary material 6 — ML tree of Graphilbum generated from the ITS sequence data [file imafungus-16-e169382-s006.png]

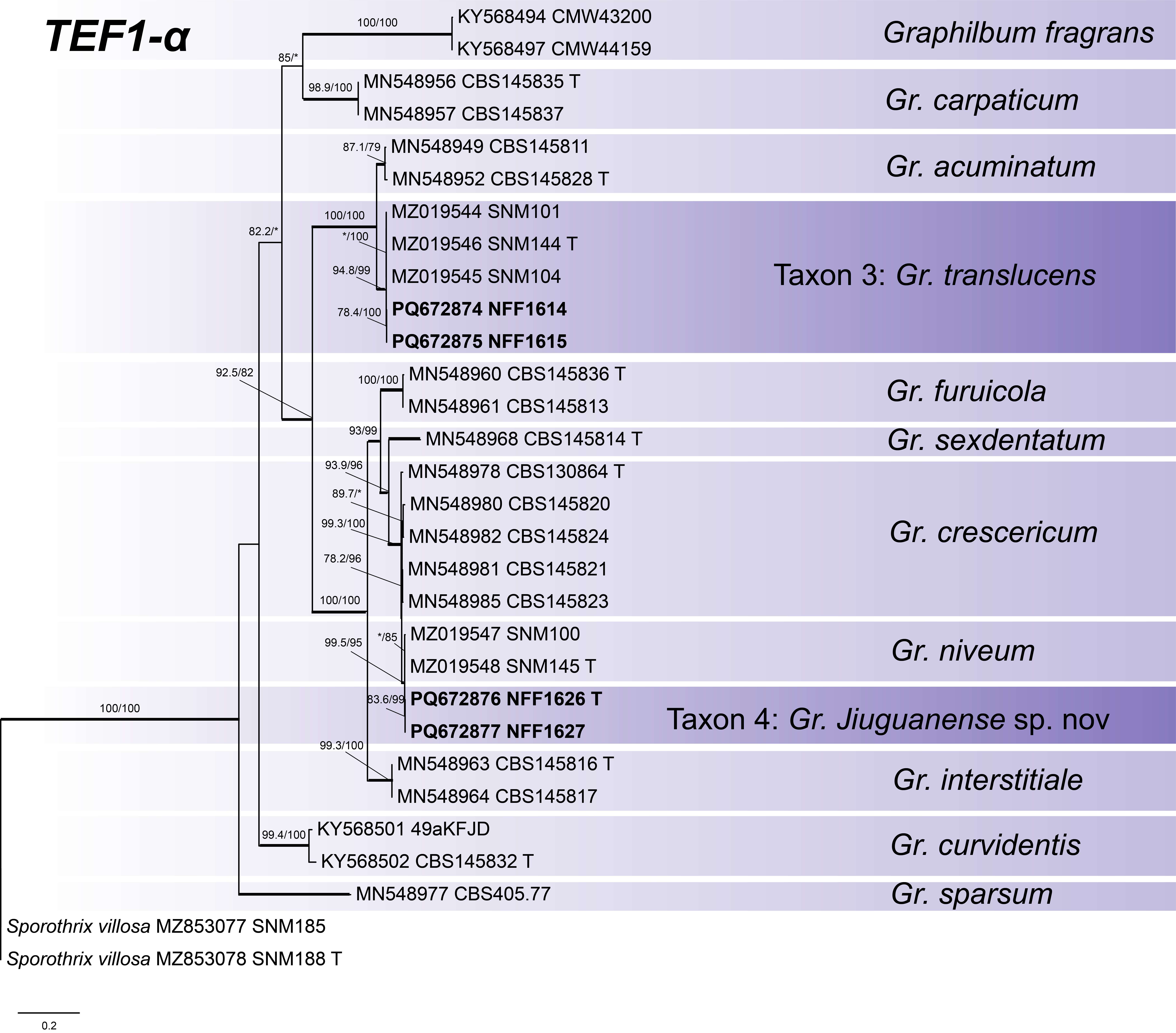

Supplement: Supplementary material 7 — ML tree of Graphilbum generated from the TEF1-α sequence data [file imafungus-16-e169382-s007.png]

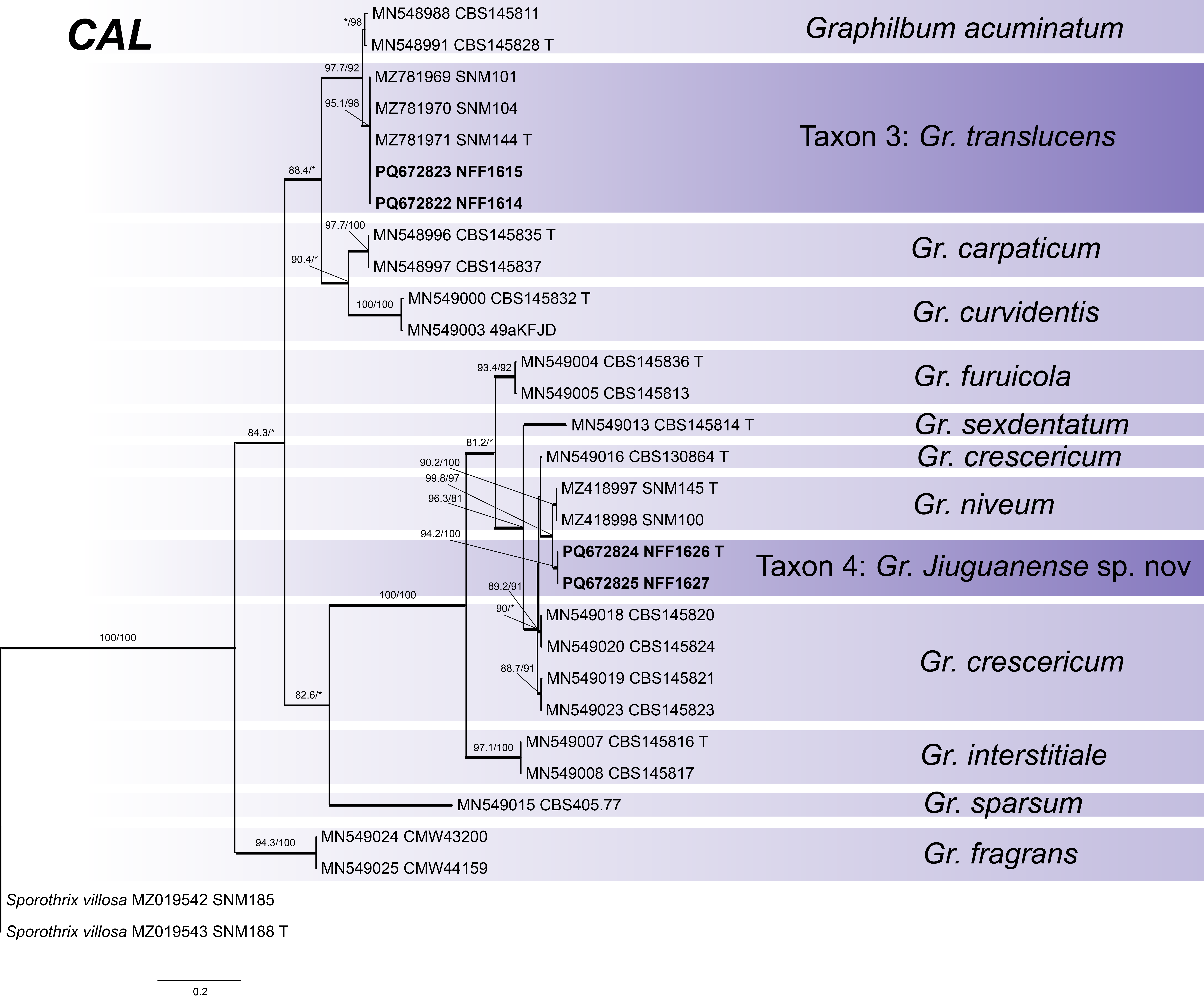

Supplement: Supplementary material 8 — ML tree of Graphilbum generated from the CAL sequence data [file imafungus-16-e169382-s008.png]

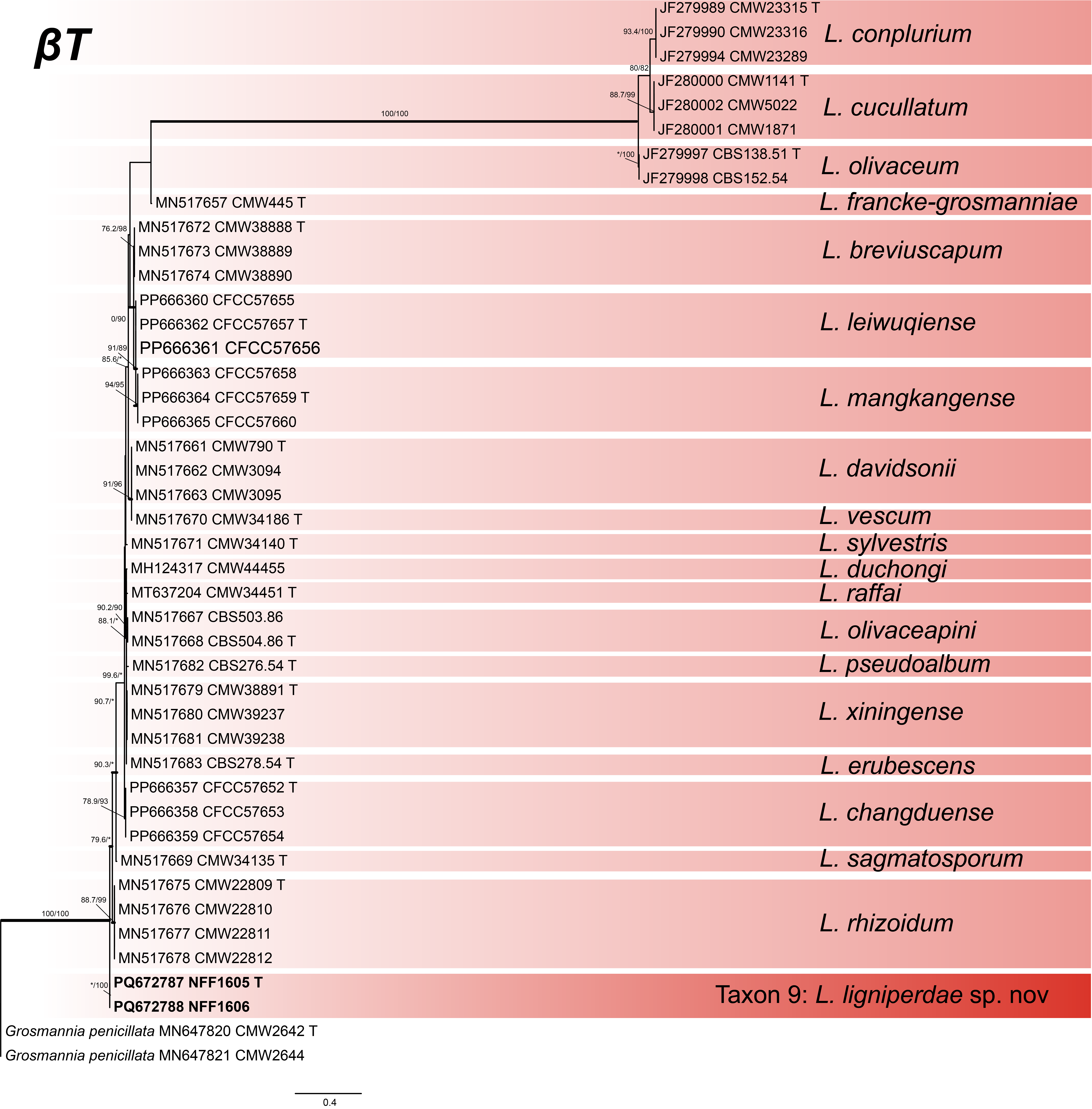

Supplement: Supplementary material 9 — ML tree of L. olivaceum complex generated from the βT sequence data [file imafungus-16-e169382-s009.png]

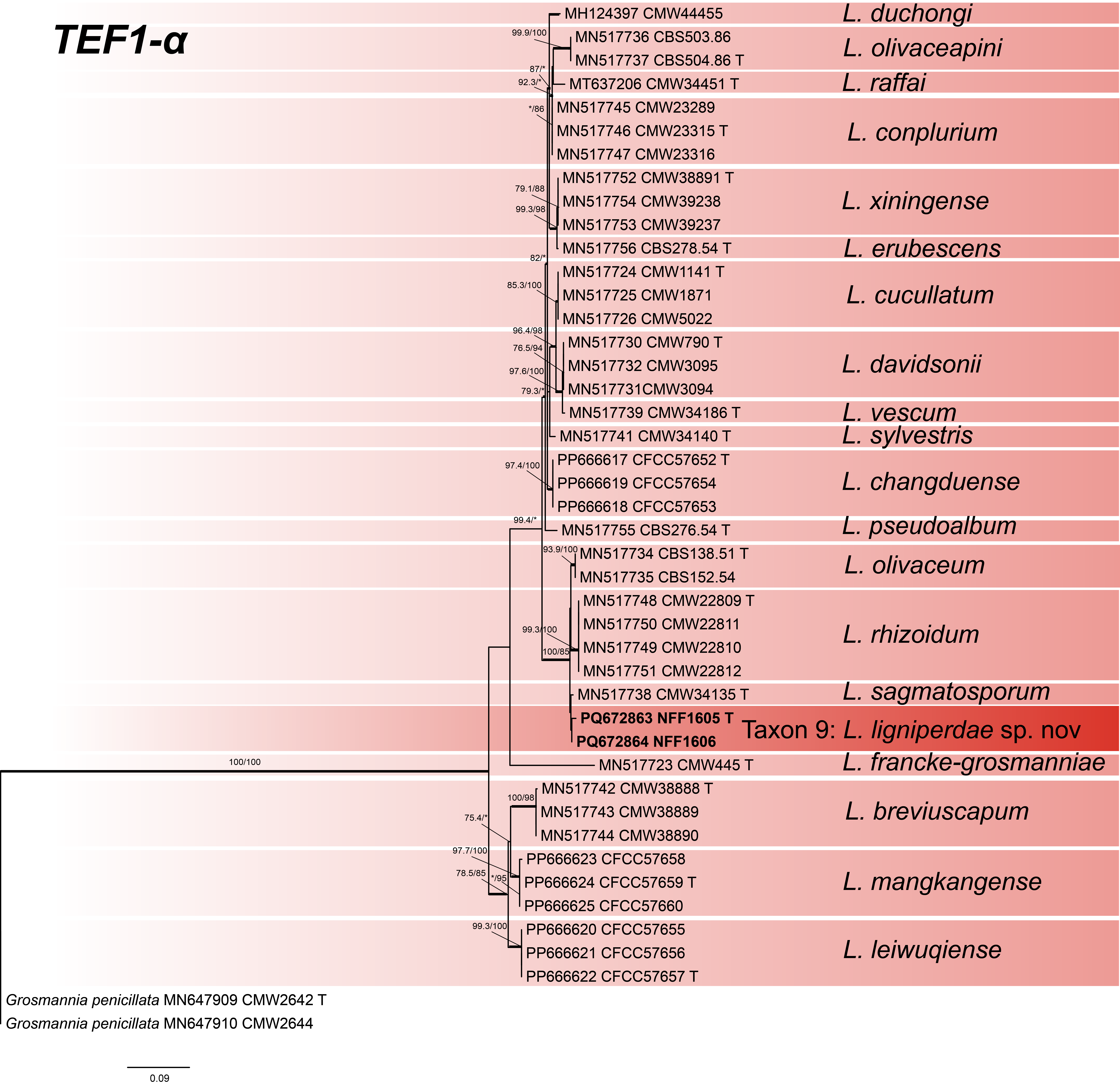

Supplement: Supplementary material 10 — ML tree of L. olivaceum complex generated from the TEF1-α sequence data [file imafungus-16-e169382-s010.png]

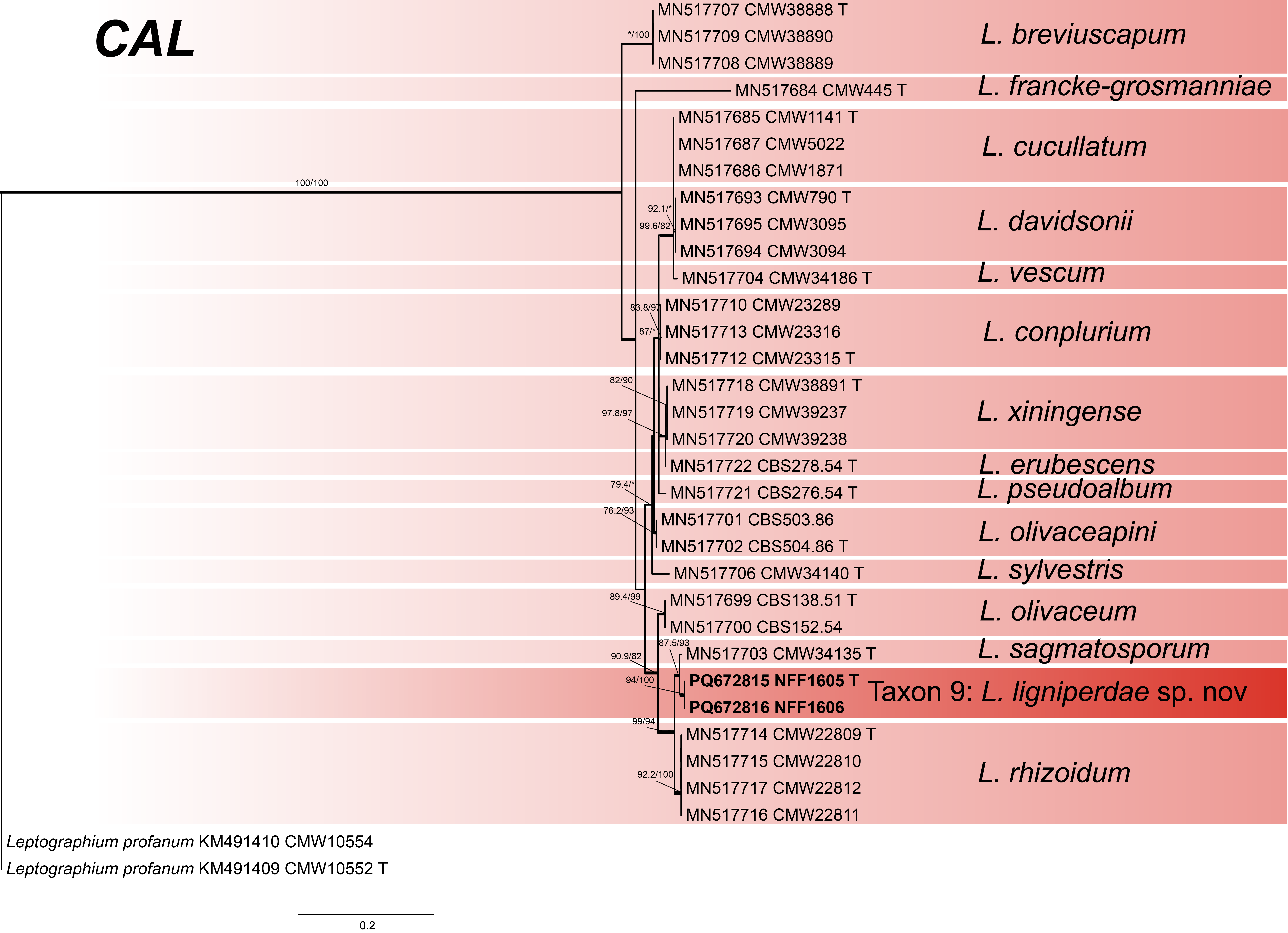

Supplement: Supplementary material 11 — ML tree of L. olivaceum complex generated from the CAL sequence data [file imafungus-16-e169382-s011.png]
